# The p97‐Nploc4 ATPase complex plays a role in muscle atrophy during cancer and amyotrophic lateral sclerosis

**DOI:** 10.1002/jcsm.13011

**Published:** 2022-05-25

**Authors:** Andrea David Re Cecconi, Mara Barone, Simona Gaspari, Massimo Tortarolo, Caterina Bendotti, Luca Porcu, Giulia Terribile, Rosanna Piccirillo

**Affiliations:** ^1^ Department of Neurosciences Mario Negri Institute for Pharmacological Research IRCCS Milan Italy; ^2^ Department of Oncology Mario Negri Institute for Pharmacological Research IRCCS Milan Italy

**Keywords:** Muscle wasting, Cancer cachexia, Amyotrophic lateral sclerosis, Protein degradation, p97‐VCP complex, Nploc4

## Abstract

**Background:**

The p97 complex participates in the degradation of muscle proteins during atrophy upon fasting or denervation interacting with different protein adaptors. We investigated whether and how it might also be involved in muscle wasting in cancer, where loss of appetite occurs, or amyotrophic lateral sclerosis (ALS), where motoneuron death causes muscle denervation and fatal paralysis.

**Methods:**

As cancer cachexia models, we used mice bearing colon adenocarcinoma C26, human renal carcinoma RXF393, or Lewis lung carcinoma, with breast cancer 4T1‐injected mice as controls. As ALS models, we employed 129/SvHsd mice carrying the mutation G93A in human SOD1. The expression of p97 and its adaptors was analysed in their muscles by quantitative real‐time polymerase chain reaction (qPCR) and western blot. We electroporated plasmids into muscles or treated mice with disulfiram (DSF) to test the effects of inhibiting p97 and nuclear protein localization protein 4 (Nploc4), one of its adaptors, on atrophy.

**Results:**

The mRNA levels of p97 were induced by 1.5‐fold to 2‐fold in tibialis anterior (TA) of all the cachectic models but not in the non‐cachectic 4T1 tumour‐bearing mice (*P* ≤ 0.05). Similarly, p97 was high both in mRNA and protein in TA from 17‐week‐old SOD1^G93A^ mice (*P* ≤ 0.01). Electroporation of a shRNA for murine p97 into mouse muscle reduced the fibre atrophy caused by C26 (*P* = 0.0003) or ALS (*P* ≤ 0.01). When we interrogated a microarray, we had previously generated for the expression of p97 adaptors, we found *Derl1*, *Herpud1*, *Nploc4*, *Rnf31*, and *Hsp90ab1* induced in cachectic TA from C26‐mice (Fold change > 1.2, adjusted *P* ≤ 0.05). By qPCR, we validated their inductions in TA of cachectic and ALS models and selected Nploc4 as the one also induced at the protein level by 1.5‐fold (*P* ≤ 0.01). Electroporation of a CRISPR/Cas9 vector against Nploc4 into muscle reduced the fibre atrophy caused by C26 (*P* = 0.01) or ALS (*P* ≤ 0.0001). Because DSF uncouples p97 from Nploc4, we treated atrophying myotubes with DSF, and found accumulated mono and polyubiquitinated proteins and reduced degradation of long‐lived proteins by 35% (*P* ≤ 0.0001), including actin (*P* ≤ 0.05). DSF halves Nploc4 in the soluble muscle fraction (*P* ≤ 0.001) and given to C26‐bearing mice limited the body and muscle weight loss (*P* ≤ 0.05), with no effect on tumour growth.

**Conclusions:**

Overall, cancer cachexia and ALS seem to display similar mechanisms of muscle wasting at least at the catabolic level. The p97‐Nploc4 complex appears to have a crucial role in muscle atrophy during these disorders and disrupting this complex might serve as a novel drug strategy.

## Introduction

p97 or VCP (valosin‐containing protein) is a hexameric ATPase complex with diverse functions in cells, from cell division to virus replication.^1^ All its actions are mediated by the p97‐mediated extraction of one or more proteins from a more complex structure that can be DNA,^2^ the contractile apparatus in muscles,^3^ or the endoplasmic reticulum (ER),^4^ for example. By consuming ATP, the p97 complex permits closeness between the substrate and the cofactor, catalyzing the reaction and ultimately facilitating the subsequent removal of the substrate by the proteasome.

Skeletal muscle is composed of multiple repetitions of highly organized compacted structures (i.e. the sarcomeres) that allow the muscle to contract. During various kinds of muscle atrophy, an accelerated and coordinated degradation of sarcomeric components of both the thick (i.e. myosin) and thin filaments (i.e. actin) occurs.^5^ Previous work showed that the myofibrils to be degraded require prior disassembly to become prone to ubiquitination and subsequent degradation by means of the proteasome.^3^, ^6^ The p97 complex seems to use energy also to render the myofibrils more loosely organized, allowing them for more effective modification by ubiquitin ligases.

p97 mutations cause multisystem genetic disorders afflicting more than one tissue, comprising skeletal muscles. These include the inclusion‐body myopathy with Paget's disease of the bone and frontotemporal dementia^7^ and amyotrophic lateral sclerosis (ALS),^8^ whose hallmarks are ubiquitin‐positive inclusions in muscle, brain and spinal cord, aberrant vacuolation, sarcomeric disorganization, muscle weakness, and functional impairment.^9^ The p97 complex exerts various intracellular functions through its ability to interact with a multitude of substrate‐recruiting and substrate‐processing adaptors.^10^ Examples of recruiting cofactors are p47 and Ufd1‐Nploc4 dimer. Nuclear protein localization protein 4 (Nploc4) forms a heterodimer with another adaptor of p97, Ufd1, binding to the N‐terminus of p97. This complex is recruited by the proteins anchored to the ER membrane and chromatin, binding the ubiquitinated proteins and facilitating their diversion towards the proteasome.^11^

Substrates bound to p97 can be further processed by ubiquitination, deubiquitination, or deglycosylation. The substrate‐processing cofactors include many ubiquitin ligases. One E3 that is especially important in muscle is Fbxo32/atrogin 1 (also named Mafbx), which may bind to p97,^12^ and adds ubiquitin molecules to the substrate, particularly during the accelerated degradation of muscle proteins that occurs during various types of atrophy, including that in cancer and most likely ALS.^13^, ^14^

Cancers and ALS all cause extensive muscle loss that culminates in cardio‐respiratory failure and early death.^15^, ^16^ Despite the different aetiology, in both cancer cachexia and in ALS, a similar set of genes may be altered (i.e. atrogenes), driving muscle wasting,^5^, ^14^ and high blood levels of inflammatory cytokines as tumour necrosis factor α (TNFα), interferon γ (IFNγ), and interleukin 6 (IL6) have been found in animal models of both disorders.^17^, ^18^ Very recently, neuromuscular junction disruption and denervation have been described in cancer cachexia as well.^19^, ^20^ Intriguingly, p97 mutations have been found in 1–2% of ALS cases,^8^ but a role of p97 in muscle wasting induced by cancer or ALS has not yet been explored.

The p97 complex participates in the degradation of muscle proteins during atrophy induced by fasting or denervation.^3^ So, we questioned whether and how it might also be involved in muscle wasting in cancer, where loss of appetite occurs, or in ALS, where progressive loss of motoneurons causes muscle denervation and subsequent fatal paralysis. The p97 complex is ubiquitous and involved in so many vital processes that its deletion is embryonic lethal.^21^ It is therefore unpractical to block p97 to cure muscle wasting, so we set out to identify which cofactor(s) of p97 is/are mostly involved in muscle wasting due to cancer and ALS. Through genetic and pharmacological approaches using *in vitro* and *in vivo* mouse models, we show that Nploc4 seems the most important interacting protein of p97 with a role in muscle atrophy associated to cancer or ALS.

Our data further indicate disulfiram (DSF), an inhibitor of Nploc4 activity, as a novel drug that may help spare muscle mass.

## Material and methods

### Cell culture and drugs

C2C12 (ATCC, Manassas, VA, USA), a myoblast cell line, was grown in Dulbecco's modified Eagle's medium (DMEM, Gibco, Waltham, MA, USA), supplemented with 10% foetal bovine serum (Euroclone, Pero, Italy) and 2 mM l‐glutamine (BioWest, Nuaillè, France), and cultured at 37°C with 5% CO_2_. Myoblasts were differentiated into myotubes when reaching 80% confluence and were cultured for 4 days in DMEM, supplemented with 2 mM l‐glutamine and 2% horse serum (Euroclone), at 37°C and 8% CO_2_. The differentiation medium was changed every 2 days. To investigate the effect of DSF on Nploc4, on the fourth day of differentiation, myotubes were treated for 24 h with vehicle (DMSO) or 1 μM DSF/1 μM Copper (Cu^2+^) in presence or not of 10 ng/mL IFNγ/TNFα (PrepoTech, Hamburg, Germany), or their combination. C26, a colon cancer cell line, was grown in DMEM supplemented with 10% foetal bovine serum and 2 mM l‐glutamine, at 37°C with 5% CO_2_. These cells were kindly donated by Prof. Colombo (Fondazione IRCCS‐Istituto Nazionale dei Tumori, Milan, Italy). LLC, a lung cancer cell line, was grown in the same conditions and kindly donated by Prof. Costelli (University of Turin, Italy). 4T1 cells were grown in Ham's F12 (Gibco) with 10% foetal bovine serum and 2 mM l‐glutamine, at 37°C with 5% CO_2._ RXF393 cells were used as in Pretto *et* *al*.^22^ Cells were not contaminated by mycoplasma.

### Mice and tumour models

C26 (10^6^ cells) and 4T1 (2 × 10^5^ cells) were injected subcutaneously into the upper right flank of male or female BALB/c mice (Harlan Laboratories, Lesmo, Italy). LLC (10^6^ cells) were similarly injected in male C57BL/6 mice (Harlan Laboratories). All the mice were 10‐week‐old at the time of injection. RXF393 cells were injected orthotopically (10^5^ cells) into the right kidney of 6‐week‐old to 8‐week‐old female NCr‐nu/nu mice (Harlan Laboratories), as in Pretto *et al*.^22^ Nude mice were maintained under specific pathogen‐free conditions and handled using aseptic procedures. Mice injected with equal volume of phosphate‐buffered saline (PBS, Gibco) served as control.

Mice were weighed the day of the injection, then every 2 days until they began to lose weight, after which they were weighed daily. In accordance with institutional guidelines, animals were killed when at least four out of five signs of distress were present (loss of mobility, kyphosis, ruffled fur, dehydration, and tremor) or when more than 20% of body weight was lost in 72 h. *In vivo* experiments were carried out in blinded conditions. C26 mice (9–10 per group) were randomized to receive DSF 50 mg/kg or vehicle every 48 h from Days 3 to 13. Hindlimb skeletal muscles and tumours were collected at sacrifice. Female transgenic SOD1^G93A^ mice expressing ∼20 copies of human mutant SOD1 with a G93A substitution on 129/SvHsd genetic background and corresponding non‐transgenic (Ntg) female littermates were used.^23^

Procedures involving animals and their care were conducted in conformity with institutional guidelines in compliance with national and international laws and policies (authorizations n° 51/2021‐PR and 814/2019‐PR). The Mario Negri Institute for Pharmacological Research IRCCS (IRFMN) adheres to the principles set out in the following laws, regulations, and policies governing the care and use of laboratory animals: Italian Governing Law (D.lgs 26/2014; Authorization n° 19/2008‐A issued 6 March 2008 by Ministry of Health); Mario Negri Institutional Regulations and Policies providing internal authorization for persons conducting animal experiments (Quality Management System Certificate‐UNI EN ISO 9001:2015‐Reg. n° 6121); the National Institutes of Health Guide for the Care and Use of Laboratory Animals (2011 edition) and European Union directives and guidelines (European Economic Community (EEC) Council Directive 2010/63/UE).

### Microarrays

We used SurePrint G3 Mouse Gene Expression Microarray Kit v2 8x60K (design 028 005 ID, Agilent Technologies, Santa Clara, CA, USA) for analysis of tibialis anterior (TA) of C26‐bearing mice, and PBS mice as control.

### Muscle electroporation with plasmids

The endotoxin‐free Maxi Prep kit (Invitrogen, Carlsbad, CA, USA) was used to purify plasmids for muscle electroporation. As in Re Cecconi *et al*.,^24^ TA was electroporated, with the animals anesthetized by inhalation of 3% isoflurane and 1% O_2_. After 14 days, muscles were dissected and weighed by an individual unaware of the experimental conditions. We used plasmids as pspCas9 (BB)‐Nploc4‐GFP (CC9 Nploc4), pspCas9 (BB)‐2A‐GFP (PX458) (as control) (Genscript), and pGIPZ encoding for shRNA for murine p97 (Open Biosystem).

### Muscle sample processing and fibre size measurements

Ten‐micrometre‐thick cryosections of electroporated muscles were obtained to measure the cross‐sectional area (CSA) of transfected and non‐transfected fibres from the same muscle, in blind conditions (ImageJ software, National Institutes of Health, Bethesda, MA, USA). The CSA in *Figure* 8 was evaluated in blind conditions with ImageJ on 10‐μm‐thick cryosections of frozen TA sections stained with wheat‐germ agglutinin (Thermo Fisher Scientific, Waltham, MA, USA).

Pictures of muscle fibres were acquired with an Olympus Microscope IX71 (×20 magnification, ×10 ocular lens, Olympus, Shinjuku, Japan) with Cell F (2.6 Build1210, Olympus, Shinjuku, Japan) imaging software for Life Science microscopy (Olympus Soft Imaging solution GmbH, Munster, Germany).

### RNA isolation from cultured cells or muscles and reverse transcription

Total RNA was isolated from cells or muscles with QIAzol Lysis Reagent (Qiagen, Hilden, Germany) and miRNeasy Kit (Qiagen). RNA concentration, purity, and integrity were measured with NANODROP 1000 (ThermoFisher Scientific) as in Re Cecconi *et al*.^24^

### Quantitative real‐time polymerase chain reaction

Total mRNA was analysed using TaqMan Mix (ThermoFisher Scientific) or the fluorescent intercalating DNA SYBR Green mix (Qiagen, Hilden, Germany). *IPO8* (*Importin 8*), *TBP* (*TATA‐binding protein*), and *GUSB* (*β‐glucuronidase*) were used as housekeeping genes. We used a 7900HT Fast Real‐Time PCR System (ThermoFisher Scientific).

### Protein extraction and western blot

Total proteins were extracted from myotubes using radioimmunoprecipitation assay (RIPA) buffer (Abcam, pH 7.5, 0.22% beta glycerophosphate, 0.18% sodium orthovanadate, 5% sodium deoxycholate, 0.38% EGTA, 1% sodium lauryl sulfate, 6.1% Tris, 0.29% EDTA, 8.8% sodium chloride, 1.12% sodium pyrophosphate decahydrate, 10% nonylphenol, ethoxylated), with the final addition of 4% sodium dodecyl sulfate (SDS) and phosphatase/protease inhibitors (Roche, Basel, Switzerland). The frozen muscle of interest was cut perpendicular to the tendon, supplemented with 20 μL/mg of RIPA buffer, plus SDS, as indicated above, and lysed with a T25 digital Ultra‐Turrax homogenizer (IKA, Staufen, Germany). The final protein concentration was determined using bicinchoninic acid assay (BCA, Pierce, Waltham, MA, USA). Then, 10–40 μg of proteins were added to 4 × Laemmli sample buffer (Biorad, Hercules, CA, USA), previously mixed with 10% β‐mercaptoethanol (Sigma, St. Louis, MO, USA) and boiled at 97°C for 7 min. Proteins were separated by 4–20% SDS‐polyacrylamide gel electrophoresis (Biorad) and transferred to a polyvinylidene difluoride membrane (GE Healthcare, Chicago, IL, USA) that was then saturated for 2 h at room tempearature in 5% bovine serum albumin or milk in a buffer of 20 mM Tris, 150 mM NaCl, and 0.1% Tween 20 (Sigma) (TBS‐T buffer). Soluble and insoluble proteins were obtained as described above, but, to clarify in which subfraction Nploc4 was segregated, we used the same buffers as done in Majera *et al*.^25^ The membrane was then incubated with the primary antibody O/N at 4°C. We used the following antibodies: anti‐vinculin (V9264, Sigma), anti‐p97/VCP (Epitomics), anti‐Ufd1 (Abcam), anti‐p47 (kindly donated by Prof. Meyer, University of Duisburg‐Essen, Germany), anti‐Nploc4 (Sigma), anti‐GAPDH (Sigma), anti‐ubiquitin clone Ubi‐1 (Millipore), and anti‐actin (Sigma). Band intensities were analysed using ImageJ software.

### Protein degradation in myotubes

Protein degradation of long‐lived proteins was measured in myotubes treated with 100 ng/mL IFNγ / 20 ng/mL TNFα in combination with vehicle (DMSO) or 0.1 or 1 μM DSF / 1 μM Cu^2+^. *In vitro* DSF and available Cu^2+^ ions form CuET (diethyldithiocarbamate‐copper complex), the active metabolite of DSF.^26^ Treated myotubes were incubated for 16 h with radiolabeled 3H‐tyrosine (2 μCi/mL; PerkinElmer, Waltham, MA, USA) to label long‐lived proteins, then processed as in Re Cecconi *et al*.^24^

### Statistical analysis

Sample size was determined by power analysis with G*Power (Version 3.1.9.6). Historical data from similar experiments previously published by us were used to estimate statistical parameters. One‐way analysis of variance (ANOVA) was used to compare multiple groups, followed by Tukey's post‐hoc test or Dunnett's multiple comparison post‐hoc test. Unpaired *t*‐test was used for comparisons of two groups. Normality of residuals was formally tested using Shapiro–Wilk test and graphically showed with Q–Q plots. Homoscedasticity was formally tested using Levene's test and graphically evaluated using histograms. If ANOVA assumptions were not satisfied, data were analysed using non‐parametric statistics: Kruskal–Wallis test followed by Dunn's multiple comparison post‐hoc test. A two‐way ANOVA was used to statistically detect differences in CSA between transfected and non‐transfected fibres and heterogeneity among animals in electroporation experiments, shown in *Figures* 3 and 6 and in data plotted in *Figure* 8. *P* values ≤ 0.05 were considered statistically significant. Mean and standard error of the mean (SEM) were used as summary statistics. Data were analysed with GraphPad Prism 9.1.1 for Windows (Graph‐Pad Software, San Diego, CA, USA) and StatView Software for Windows (SAS StatView for Windows, Redmond, WA, USA).

## Results

### The expression of p97 increases in atrophied tibialis anterior from mice with cancer cachexia or amyotrophic lateral sclerosis and its inhibition is beneficial

When cells of colon adenocarcinoma C26 or LLC or rare human renal carcinoma RXF393 are injected subcutaneously (C26 and LLC) or orthotopically (RXF393) in mice, they cause body weight loss to different extents, muscle depletion, and early death (i.e. cachexia).^22^, ^24^

**Figure 1:**
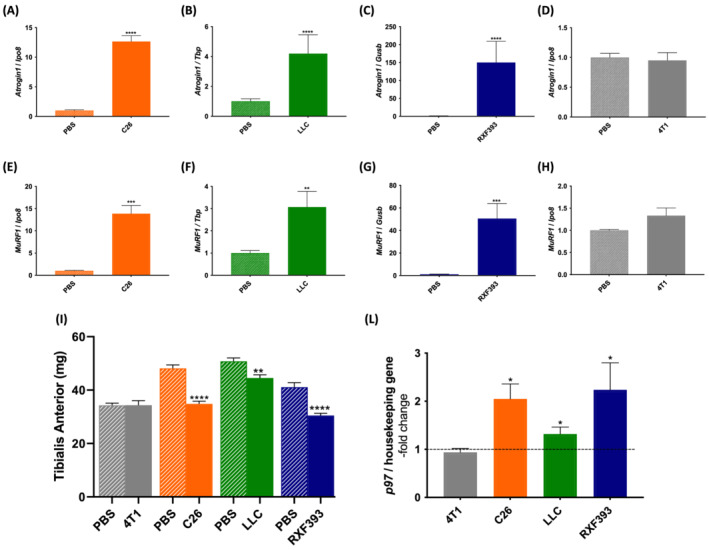
The mRNA levels of p97 rise in tibialis anterior only from cancer cachexia mouse models. The mRNA levels of *Fbxo32/atrogin 1* and *MuRF1* were measured with qPCR in tibialis anterior (TA) of C26‐ (*A*, *E*), LLC‐ (*B*, *F*), RXF393‐ (*C*, *G*), and 4T1‐bearing mice (*D*, *H*). PBS‐injected mice were used as control. Mice were sacrificed and muscle dissected after 14 (C26), 20–25 (LLC), 23 (RXF393), and 25 (4T1) days from tumour injection. C26 *n* = 8–14, LLC *n* = 8–10, RXF393 *n* = 6–7, and 4T1 *n* = 5. *Importin 8 (Ipo8*), *Tata‐binding protein* (*TBP*) and *β‐glucuronidase* (*GUSB*) were used as housekeeping genes. The weights of the TA from PBS, 4T1, C26, LLC, and RXF393 mice are shown in (*I*). 4T1 *n* = 5, C26 *n* = 8–14, LLC *n* = 10, and RXF393 *n* = 6–7. The mRNA levels of p97 in TA of 4T1, C26, LLC, and RXF393 mice were measured by qPCR and plotted as the ‐fold changes over PBS‐mice (dotted line) (L). 4T1 *n* = 5, C26 *n* = 8–14, LLC *n* = 8–10, and RXF393 *n* = 6–7. *Ipo8*, *TBP*, and *GUSB* were used as housekeeping genes. Results are plotted as mean ± SEM. Unpaired *t*‐test was done for each condition compared with its own PBS, *P ≤ 0.05, **P ≤ 0.01, ***P ≤ 0.001, and ****P ≤ 0.0001.

To confirm that muscles from these cancer‐bearing mice had atrophy typical of cachexia, we used quantitative real‐time polymerase chain reaction (qPCR) to analyse the muscle mRNA expression of *Fbxo32/atrogin 1* and *MuRF1*, the main ubiquitin ligases involved in protein degradation during cancer‐mediated muscle atrophy.^13^ In the TA from cachectic C26‐, LLC‐, and RXF393‐bearing mice, *Fbxo32/atrogin 1* was up‐regulated about 12‐fold, 4‐fold, and 150‐fold, respectively (*Figure* 1A–1C). Similarly, *MuRF1* was found induced about 12‐fold, 3‐fold, and 50‐fold, respectively (*Figure* 1E–1G), compared with PBS‐injected mice. Importantly, neither *Fbxo32/atrogin 1* nor *MuRF1* were induced in the non‐atrophying TA of breast cancer 4T1‐bearing mice that did not present cachexia (*Figure* 1D, 1H, and 1I). Accordingly, C26, LLC, and RXF393 carriers displayed muscle wasting, confirmed by reductions in the weight of their TA compared with PBS‐injected mice, while 4T1 carriers showed no muscle atrophy (*Figure* 1I).

In these mouse models, we also measured the mRNA levels of *p97*, which is up‐regulated in muscles atrophied because of denervation or fasting.^3^ *p97* was found up‐regulated about 1.5‐fold to 2‐fold only in the TA of the cachectic C26, LLC, and RXF393 mice, but not in the non‐cachectic 4T1 mice (*Figure* 1L), supporting p97 up‐regulation as a hallmark of muscle wasting. Because also the gastrocnemius was found atrophied in all the above‐mentioned models and not in 4T1 carriers, we measured the mRNA levels of *Fbxo32/atrogin 1*, *MuRF1*, and *p97* also in this other type of muscle and confirmed the findings found in TA (Supporting Information, *Figure* S1).

**Figure 2:**
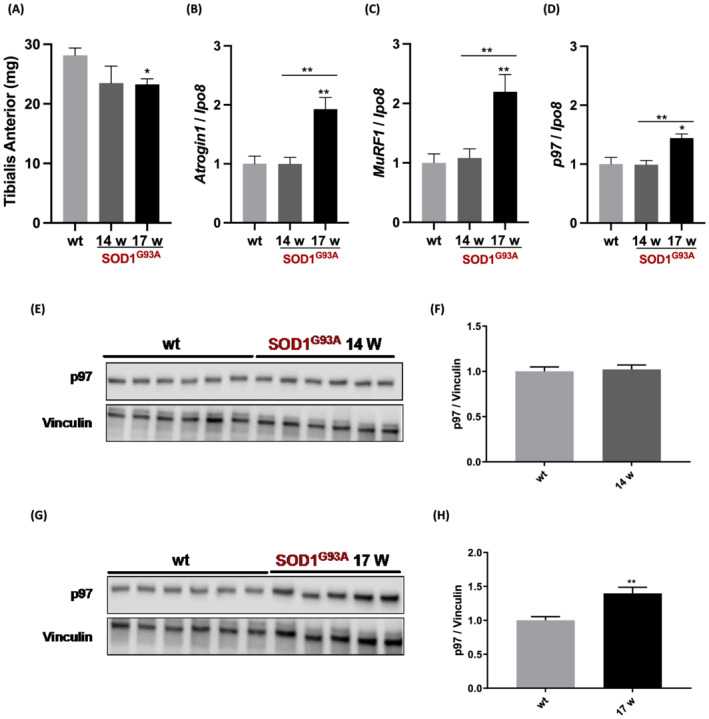
The expression of p97 rises in atrophying tibialis anterior of symptomatic SOD1^G93A^ mice. The weights of the TA from 129/SvHsd WT or SOD1^G93A^ mice are shown in (*A*). *Fbxo32/atrogin 1* (*B*), *MuRF1* (*C*), and *p97* (*D*) mRNA levels were evaluated in TA muscles of SOD1^G93A^ mice at 14 and 17 weeks of age, and compared with 14‐week‐old wild‐type mice (WT) using qPCR. 129/SvHsd WT mice served as Ntg controls. *N* = 5–6. *Ipo8* was used as housekeeping gene. TA muscles of SOD1^G93A^ mice were analysed by WB for p97 (*E*, 14‐week‐old mice; *G*, 17‐week‐old mice), and the band quantitation was plotted, *n* = 5–6 (*F*, *H*). Vinculin was used as internal loading control. 129/SvHsd WT mice were used as controls. Results are plotted as mean ± SEM, one‐way ANOVA with post‐hoc Tukey's multiple comparison test (*A*–*D*) or unpaired *t*‐test (*F*, *H*).*P ≤ 0.05, **P ≤ 0.01.

The p97 protein was mutated in a small subset of ALS patients,^8^ indicating a link between this protein and the disease. We found p97 up‐regulated in denervated muscles upon cutting the sciatic nerve,^3^ somehow recapitulated in ALS with motoneuron death.^23^ We therefore measured *Fbxo32/atrogin 1*, *MuRF1*, and *p97* transcripts also in TA from 129/SvHsd SOD1^G93A^ mice, a ‘fast progressor’ ALS mouse model. It presents a significant neuromuscular junction denervation at 14 weeks of age, at the onset of symptoms, and rapidly progresses to prominent symptomatic stage at 17 weeks,^23^ when the TA displays atrophy (*Figure* 2A). Interestingly, the mRNA levels of *Fbxo32/atrogin 1*, *MuRF1*, and *p97* were all induced about 1.5‐fold to 2‐fold in atrophied TA of SOD1^G93A^ mice at 17 weeks of age, while they were unchanged at 14 weeks (*Figure* 2B–2D). The protein content of p97 was also unchanged at 14 weeks (*Figure* 2E–2F) but was raised about 50% in TA of SOD1^G93A^ mice at 17 weeks of age (*Figure* 2G–2H), confirming the mRNA expression data. Notably, similar data were found in the atrophied gastrocnemius of SOD1^G93A^ mice (*Figure* S2A and S2B), where the protein content of p97 raised by 3‐fold at 17 weeks of age (*Figure* S2C–S2F).

To understand if the increased p97 in muscles could be due to reduced food intake as we previously found in mice upon fasting,^3^ we measured the food eaten over time by most of the models analysed (*Figure* S3). While C26‐carriers ate less than PBS‐mice (*Figure* S3A), LLC carriers did not display signs of anorexia as well as SOD1^G93A^ mice until 17 weeks of age, when they started to eat less than their healthy counterparts (*Figure* S3C–S3D). These data may suggest that the increased p97 found in muscles may not be due only to reduced food intake but mainly to the disease progression.

Pax4 is one of the transcription factors driving p97 expression,^6^ so we questioned whether it could be induced in these muscles at times when p97 was enhanced. We did not see any increase in the expression of Pax4 in muscles from either of the two disease models (*Figure* S4A–S4F), possibly excluding Pax4‐mediated induction of p97. However, the p97 complex seems enhanced in widely differing types of muscle wasting.

**Figure 3:**
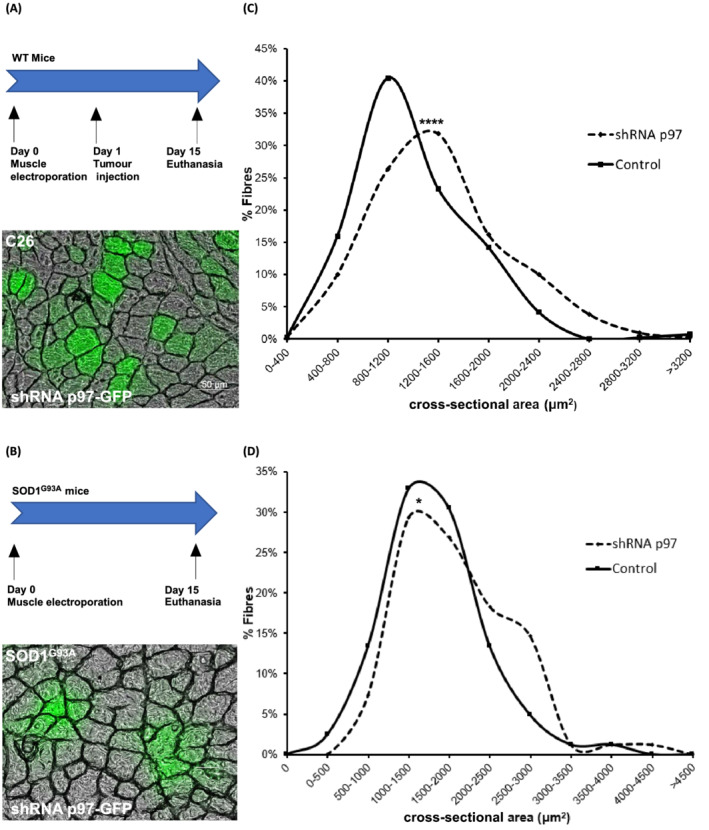
Silencing p97 preserves the fibre area of atrophying muscles of mice with C26‐induced cachexia or ALS. Experimental schedule and representative image of a transverse section of fibres electroporated with shRNAp97‐GFP carrying plasmid in C26‐bearing BALB/c mice (*A*) or 129/SvHsd SOD1^G93A^ ones (*B*) are shown. Scale bar, 50 μm. Frequency histograms showing the distribution of cross‐sectional areas of muscle fibres of TA transfected or not with the shRNAp97‐GFP carrying plasmid in C26‐mice (*C*) and 129/SvHsd SOD1^G93A^ ones (*D*). The area of non‐transfected fibres is indicated as control (*C*, *D*). A total of 420 shRNAp97‐GFP‐expressing fibres and non‐electroporated ones were analysed in (*C*) for a total of 9 mice and 82 in (*D*) for a total of 7 mice. Differences were found between shRNAp97‐GFP‐expressing fibres and controls. A statistically significant difference without heterogeneity among animals was detected in (*C*) (*P* value for difference = 0.0003; *P* value for heterogeneity = 0.53). A statistically significant difference without heterogeneity among animals was detected in (*D*) (*P* value for difference = 0.011; *P* value for heterogeneity = 0.32).

To test whether inhibiting p97 in muscles resulted in fibre preservation during C26 growth, we electroporated the TA with a shRNAp97‐carrying plasmid that we previously validated.^3^ The next day, we injected subcutaneously C26 cells, and after 14 days, we euthanized the mice and collected their muscles. The frequency histogram displaying the distribution of the CSA of muscle fibres shows that shRNAp97‐expressing fibres are bigger than the adjacent non‐electroporated ones within the same cachectic muscle (*Figure* 3A and 3C). The same was done in TA of SOD1^G93A^ mice, electroporated as above with a shRNAp97‐carrying plasmid at 13 weeks (before symptoms appeared) and euthanized 2 weeks later. Again, shRNAp97‐expressing fibres were slightly bigger than non‐transfected ones in the same diseased muscle (*Figure* 3B and 3D). Notably, the expression of shRNAp97‐carrying plasmid in healthy mice did not alter the fibre sizes of mice of both strains (BALB/c and 129/SvHsd) if compared with pGIPZ (empty vector)‐expressing fibres of the contralateral muscles (*Figure* S5A–S5D).

Therefore, inhibiting p97, at least in skeletal muscles, seems to be sufficient to limit muscle atrophy caused by cancer or ALS.

### Nploc4 as the most induced p97 cofactor in atrophying muscles during cancer or ALS

Because inhibiting p97 systemically is lethal,^21^ we set out to discover the p97 adaptor(s) that could be the preferred partner(s) of p97 in the enhanced proteolysis during cancer‐ or ALS‐related muscle atrophy.

**Figure 4:**
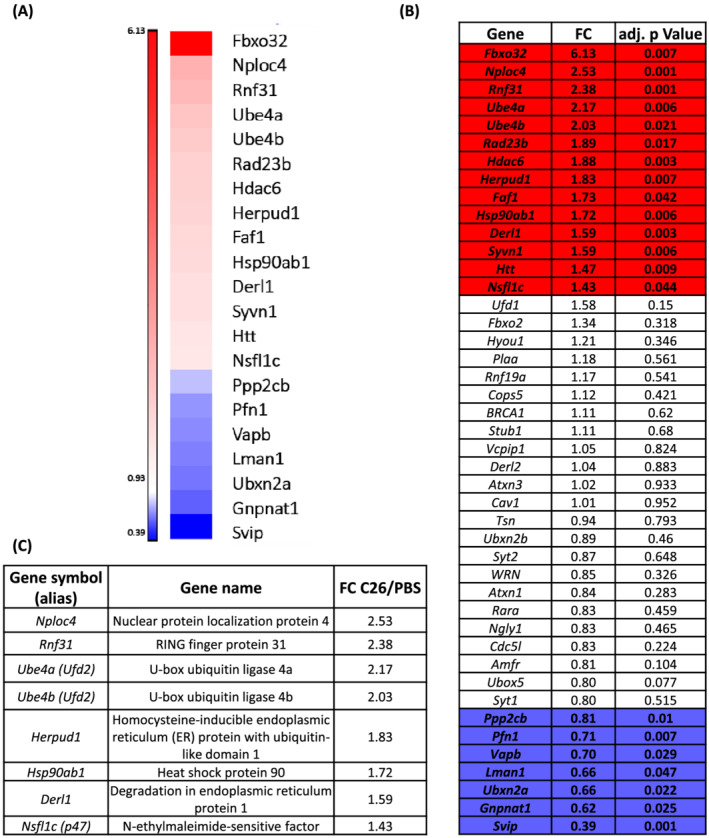
Gene expression of p97 adaptors in muscles from C26‐carrying mice. Microarray analysis of TA muscles from 10‐week‐old male mice, showing atrophy because of the growth of C26, with about 13.5% body weight loss (BWL) and 34–35% muscle weight loss was performed. Age and sex‐matched mice injected with PBS served as controls (*A*, *B*). SurePrint G3 mouse gene expression microarray kit v2 8x60K (Agilent Technologies) was used. Only the genes with an induction ≥ 1.2 (in red) or with a reduction ≤ 0.85 (in blue), with Bonferroni‐adjusted *P* ≤ 0.05 are highlighted (B). Multiple testing correction. A summary of the p97 cofactors induced and that may be involved in cancer cachexia is shown (*C*).

We went through the literature and prepared a list of p97‐interacting proteins that were *bona fide* substrate‐recruiting or substrate‐processing adaptors of p97 but not at all its substrates. We screened out 44 listed proteins from our microarray gene datasets on cachectic TA muscles from C26‐mice compared with PBS‐mice, as in Re Cecconi *et al*.^24^ Twenty‐three genes were unchanged, 14 up‐regulated (Fold change > 1.2), and only 7 down‐regulated (Fold change < 0.85) in muscles with C26‐induced cachexia (adjusted *P* value ≤ 0.05). Among the up‐regulated genes encoding for p97 cofactors, we found *Fbxo32*/*atrogin 1*, *Nploc4*, *Rnf31*, *Ube4a*/*Ufd2*, *Ube4b*/*Ufd2*, *Rad23b*, *Hdac6*, *Herpud1*, *Faf1*, *Hsp90ab1/Hsp90*, *Derl1*, *Syvn1*, *Htt*, and *Nsfl1c*/*p47* (*Figure* 4A, 4B, and 4C). Instead, *Ppp2cb*, *Pfn1*, *Vapb*, *Lman1*, *Ubxn2a*, *Gnpnat1*, and *Svip* were down‐regulated (*Figure* 4B).

**Figure 5:**
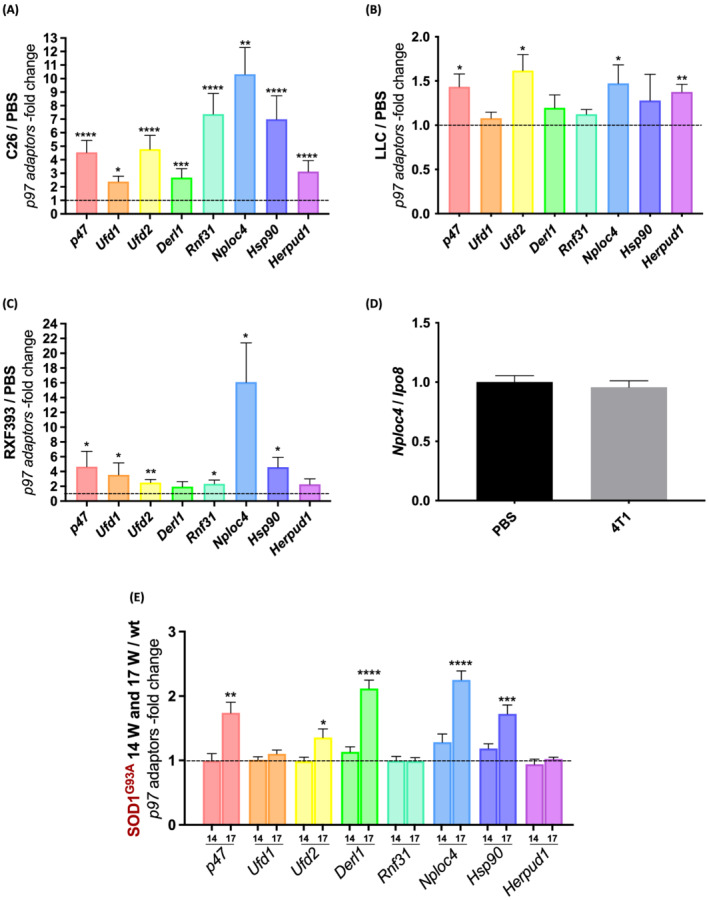
Nploc4 is the p97 cofactor most induced in atrophying tibialis anterior from either cancer cachexia models or SOD1^G93A^ mice. The mRNA levels of p97 adaptors in TA of C26 *(A*), LLC (*B*), and RXF393 mice (*C*) were measured by qPCR and plotted as ‐fold changes over PBS‐mice (dotted line). PBS‐mice were used as controls. C26 *n* = 12, LLC *n* = 10, and RXF393 *n* = 6–7. *Ipo8* and *TBP* served as housekeeping gene for (*A–C*). *Nploc4* mRNA levels were evaluated in non‐cachectic TA muscles of 4T1‐mice by qPCR (*D*), *n* = 5. Unpaired *t*‐test, not significant. The mRNA levels of p97 adaptors were measured by qPCR in TA muscles of 14‐week‐old or 17‐week‐old 129/SvHsd SOD1^G93A^ mice and shown as the ‐fold changes over healthy or WT mice (dotted line) (*E*), *n* = 5–6. *Ipo8* was used as housekeeping gene. Results are plotted as mean ± SEM. Unpaired *t*‐test was done for each condition compared with its own PBS, *P ≤ 0.05, **P ≤ 0.01, ***P ≤ 0.001, and ****P ≤ 0.0001.

Excluding *Fbxo32*/*atrogin 1* and *Hdac6* for which a major role in muscle wasting is already clear,^13^, ^27^ we selected among them some of the most induced genes to validate in qPCR (summary in *Figure* 4C). In the cachectic TA of BALB/c mice bearing C26, we confirmed *Nploc4* to be the most induced (about 10‐fold), while the other adaptors (*p47*, *Ufd1*, *Ufd2*, *Derl1*, *Rnf31*, *Hsp90*, and *Herpud1*) were up‐regulated about 2‐fold to 7‐fold with respect to healthy mice (*Figure* 5A). In the TA of LLC‐bearing mice, a model of cancer cachexia but in C57BL/6 background, the mRNA levels of *p47*, *Ufd2*, *Nploc4*, and *Herpud1* were all induced by about 50%, while *Ufd1*, *Derl1*, *Rnf31*, and *Hsp90* did not change (*Figure* 5B).

To further characterize the muscle expression of p97 adaptors in cachexia, we moved to immunodeficient mice with the human renal carcinoma RXF393 implanted orthotopically, more resembling the human pathology. *Nploc4* was strongly up‐regulated—about 16‐fold—in this cachectic TA, while other adaptors were induced about 2‐fold to 4‐fold, except *Derl1* and *Herpud1*, which did not change at all (*Figure* 5C). Notably, there was no *Nploc4* induction in TA from non‐cachectic female mice bearing 4T1, supporting Nploc4 induction as a hallmark of muscle wasting during cachexia and not in cancer in general (*Figure* 5D). Of note, similar data were found when the mRNA levels of all these adaptors were measured in the atrophied gastrocnemius in all the models suffering from cancer cachexia (C26, LLC, and RXF393) (*Figure* S6A–S6C) or controls (4T1) (*Figure* S6D), indicating that our data are reproducible and not limited to a single type of muscle. Furthermore, we measured Nploc4 and p97 in gastrocnemius of another model of cancer cachexia we routinely use, mice carrying MCG101 (methylcholanthrene‐induced sarcoma). Again, we found elevated levels of both *Nploc4* and *p97* (*Figure* S7A and S7B) at times when *Fbxo32/atrogin 1* and *MuRF1* were induced (*Figure* S7C and S7D).

To learn whether *Nploc4* was also induced in muscle in a tumour‐free model of muscle atrophy, as ALS, by qPCR, we analysed the expression of selected p97 cofactors in TA muscles from 14‐week‐old or 17‐week‐old SOD1^G93A^ mice. No cofactor changed in TA at the onset of the pathology, but *p47*, *Ufd2*, *Derl1*, *Nploc4*, and *Hsp90* were enhanced about 1.5‐fold to 2‐fold at 17 weeks of age (*Figure* 5E). Again, *Nploc4* was among the most induced cofactors.

**Figure 6:**
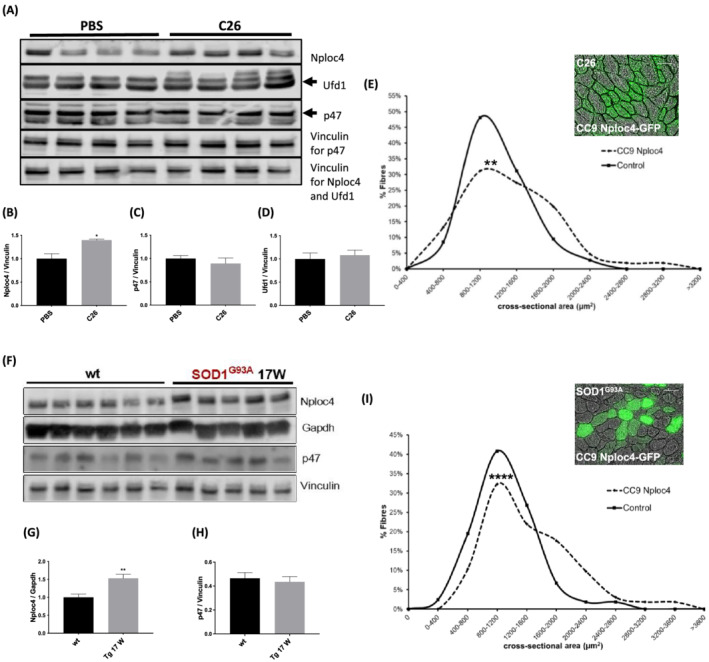
Silencing Nploc4 preserves the fibre area of atrophying muscles of mice with C26‐induced cachexia or ALS. TA muscles of C26‐bearing mice were analysed by WB for Nploc4, p47, and Ufd1 (*A*). The band quantitations are plotted, *n* = 4 (*B*–*D*). PBS‐injected mice were used as controls and vinculin, as internal loading control. Frequency histograms showing the distribution of cross‐sectional areas of muscle fibres of TA of C26 bearing‐mice transfected or not (control) with CRISPR/Cas9 Nploc4 vector are shown in (*E*), *n* = 106 fibres for a total of 7 mice. A statistically significant difference between transfected and non‐transfected fibres with heterogeneity among animals was detected in (*E*) (*P* value for difference = 0.010; *P* value for heterogeneity = 0.026). TA muscles of 17‐week‐old 129/SvHsd SOD1^G93A^ mice were analysed by WB for Nploc4 and p47 (*F*). The related band quantitations are plotted, *n* = 5–6 (*G*, *H*). Vinculin and GAPDH were used as internal loading controls and 14 week‐old 129/SvHsd WT mice as controls. Frequency histograms showing the distribution of cross‐sectional areas of muscle fibres of TA of SOD1^G93A^ mice transfected or not (control) with CRISPR/Cas9 Nploc4 vector are shown in (*I*), *n* = 164 fibres for a total of five mice. A representative image of a transverse section of fibres electroporated with CRISPR/Cas9 Nploc4 plasmid (CC9 Nploc4‐GFP) in C26‐bearing BALB/c mice (*E*) or 129/SvHsd SOD1^G93A^ ones (*I*) are shown. Scale bar, 50 μm. Results are plotted as mean ± SEM. *P ≤ 0.05, **P ≤ 0.01, unpaired *t*‐test (*B*–*D*, *G*–*H*). A statistically significant difference between transfected and non‐transfected fibres with heterogeneity among animals was detected in (*I*) (*P* value for difference ≤ 0.0001; *P* value for heterogeneity = 0.006).

Then, we evaluated the protein expression of Nploc4 and some of the other adaptors in TA of C26‐bearing mice and SOD1^G93A^ mice. Nploc4 protein content was raised more than 30% in TA muscle of C26‐mice (*Figure* 6A and 6B), while p47 (*Figure* 6A and 6C) and Ufd1 did not change (*Figure* 6A and 6D). Similarly, Nploc4 protein content was also raised by about 50% in TA muscle of SOD1^G93A^ mice at the symptomatic stage of the disease (*Figure* 6F and 6G), while p47 did not (*Figure* 6F and 6H), and similarly it occurred in the atrophied gastrocnemius of SOD1^G93A^ mice (*Figure* S8A–S8E). Again, Nploc4 protein content seemed to display an increased trend also in mouse muscles of carriers of other cachexia‐promoting tumours (RXF393 and LLC) at times when MuRF1 protein accumulated (*Figure* S9A–S9I), but not of tumours unable to cause cachexia (4T1) (*Figure* S10A–S10E).

Our data clearly show that among the p97 cofactors, Nploc4 is the one most induced in atrophying muscles from three unrelated models of cancer cachexia (C26, LLC, and RXF393) and from SOD1^G93A^ mice, even though this induction did not anticipate muscle depletion.

### Silencing Nploc4 can delay muscle atrophy caused by colon cancer or ALS

Because Nploc4 was the only p97 cofactor, among those analysed, induced both at mRNA and protein levels in the wasted muscles, we employed a CRISPR/Cas9 Nploc4‐encoding plasmid to test whether down‐regulation of Nploc4 in muscles resulted in fibre preservation during C26 growth.

Preliminarily, we transfected myoblasts with the CRISPR/Cas9 Nploc4‐encoding plasmid and T7E1 cleavage assay showed clear editing of the target gene (*Figure* S11), indicating that such plasmid but not the control vector (CC9) carries a guide that can edit the target gene. We then electroporated CRISPR/Cas9 Nploc4‐encoding plasmid in TA of C26‐bearing mice and after 14 days the mice were euthanized and muscles were analysed. Intriguingly, fibres transfected *in vivo* for CRISPR/Cas9 Nploc4 had a bigger mean CSA than non‐electroporated ones in the same muscle (*Figure* 6E).

To verify whether restraining Nploc4 in muscles results in fibre preservation also in a non‐cancerous model of muscle atrophy where it was induced, we electroporated CRISPR/Cas9 Nploc4‐encoding plasmid in TA of SOD1^G93A^ mice at 15 weeks of age and after 14 days, at the symptomatic stage, the mice were sacrificed and muscles were analysed. Like in C26‐mice, mean CSA of fibres transfected *in vivo* for CRISPR/Cas9 Nploc4 appeared bigger than that of negative and adjacent ones within the same muscle (*Figure* 6I). Notably, the expression of CRISPR/Cas9 Nploc4‐encoding plasmid in healthy mice did not alter fibre sizes of mice of both strains if compared with areas of fibres expressing an empty vector in the contralateral muscle (*Figure* S12A–D).

These data illustrate the importance of Nploc4 in muscle wasting because silencing it preserves the fibre area of atrophying muscles of mice suffering either from C26‐induced cachexia or ALS.

### Disulfiram, a Nploc4 inhibitor, has anti‐catabolic action on TNFα/IFNγ‐atrophying myotubes

Nploc4 is targeted and aggregated by the metabolite of DSF/Cu^2+^, the bis‐diethyldithiocarbamate‐copper complex (CuET)^25^ that inhibits Nploc4 function.^25^, ^26^

**Figure 7:**
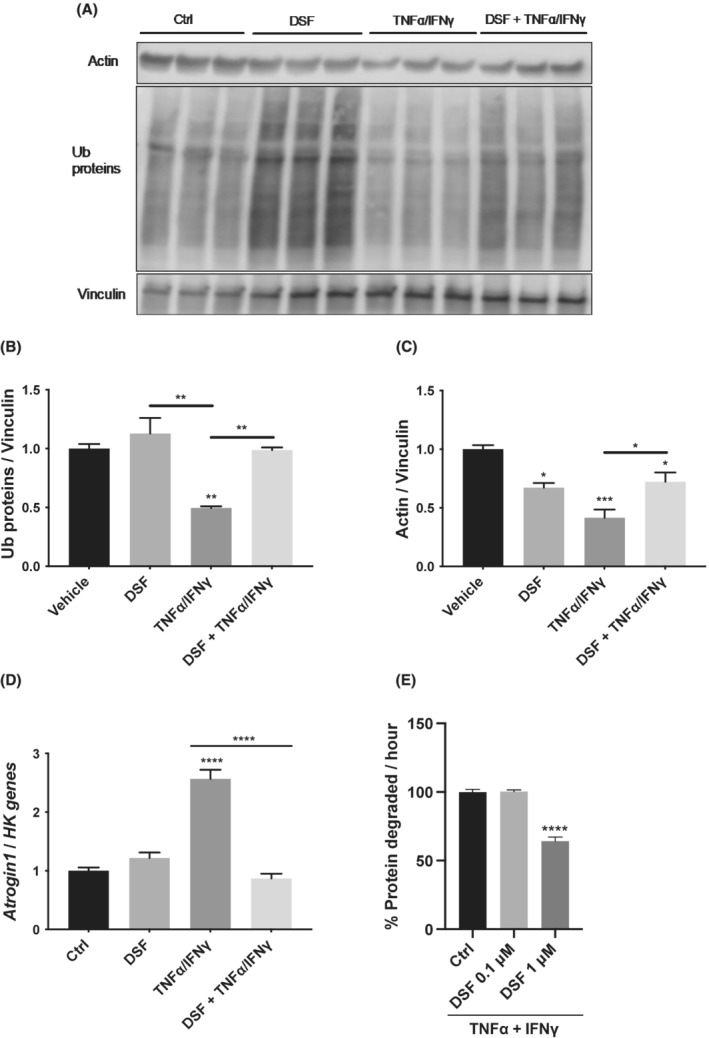
Disulfiram exerts anti‐catabolic action on atrophying myotubes. Myotubes treated for 24 h with vehicle or 1 μM DSF / 1 μM Cu^2+^ in combination with vehicle or 10 ng/mL IFNγ/TNFα were analysed by WB for poly and monoubiquitinated proteins and actin (*A*), *n* = 3. The band quantitations are plotted in (*B*) and (*C*). Vinculin was used as loading control. The mRNA levels of *Fbxo32/atrogin 1* were evaluated by qPCR in (*D*), *n* = 3. *Ipo8*, *TBP*, and *GUSB* were used as housekeeping (HK) genes. Protein degradation of long‐lived proteins was measured in myotubes treated for 24 h with 100 ng/mL IFNγ / 20 ng/mL TNFα with vehicle (Ctrl) or 0.1 or 1 μM DSF / 1 μM Cu^2+^ (*E*). Results are plotted as mean ± SEM. One‐way ANOVA with post‐hoc Tukey's multiple comparison test (*B*–*E*), *P ≤ 0.05, **P ≤ 0.01, ***P ≤ 0.001, ****P ≤ 0.0001.

To further dissect the possible effects and mechanisms of DSF on Nploc4 in atrophic pathways, we analysed protein catabolism in C2C12 myotubes exposed to TNFα/IFNγ (a condition causing *in vitro* atrophy by inducing protein degradation^28^) and treated with DSF/Cu^2+^ for 24 h. First, we ran sulforhodamine B assays to see whether DSF was toxic in differentiated myotubes treated for 24 h with doses from 0.1 to 10 μM. Only the highest dose reduced cell viability by about 80% (*Figure* S13). Among various ways to induce atrophy *in vitro*, we chose TNFα/IFNγ treatment because it resulted in increased *Nploc4* expression, recapitulating *in vivo* models (*Figure* S14). So, we treated myotubes with 1 μM DSF and found that DSF accumulated ubiquitinated (Ub) proteins and even TNFα/IFNγ‐treated myotubes had low Ub protein contents that could be raised by DSF (*Figure* 7A and 7B). Because the antibody used crossreacted with either polyUb proteins or monoUb ones that are roughly half of total Ub conjugates, these data indicate that (poly)ubiquitination and proteolysis can be hampered by DSF also in atrophic conditions.

Since we previously found that actin is degraded through p97‐Ufd1 complex in myotubes,^3^ we measured its protein content. Atrophying TNFα/IFNγ‐treated myotubes had a lower actin content that could be somewhat increased by co‐treatment with DSF (*Figure* 7A and 7C). Unexpectedly, as soon as the polyubiquitinated proteins accumulated with DSF, the induction of *Fbxo32/atrogin 1* in TNFα/IFNγ‐treated myotubes was completely prevented (*Figure* 7D), possibly suggesting that some inhibitor(s) of *Fbxo32/atrogin 1* expression could be spared by DSF.

To further investigate the possible role of DSF in myotube protein catabolism, we measured the rates of degradation of long‐lived proteins in myotubes treated with 20 ng/mL TNFα / 100 ng/mL IFNγ, in combination with vehicle or 0.1 or 1 μM DSF. Unlike 0.1 μM, 1 μM DSF reduced the effect of TNFα/IFNγ, resulting in a lower percentage of proteins degraded per hour (64%) compared to the vehicle‐treated cells (*Figure* 7E).

Altogether, by accumulating (poly)ubiquitinated substrates, partially preserving actin and reducing the gene expression of *Fbxo32/atrogin 1*, DSF appears to prevent overall proteolysis, in accordance with its ability to inhibit the proteasomal degradation pathway by altering the p97‐Nploc4 complex.

### Disulfiram prevents muscle wasting in C26‐bearing mice

Prompted by the results obtained *in vitro*, we questioned whether DSF could counteract cancer cachexia in C26‐bearing mice. In order to establish the minimal dose and the dosing schedule to inhibit the p97‐Nploc4 complex in muscles, we tested three doses already used by others in mice^26^: 50, 100, and 200 mg/kg, euthanizing the mice 24 or 48 h after a single oral (gavage) dose of DSF.

**Figure 8:**
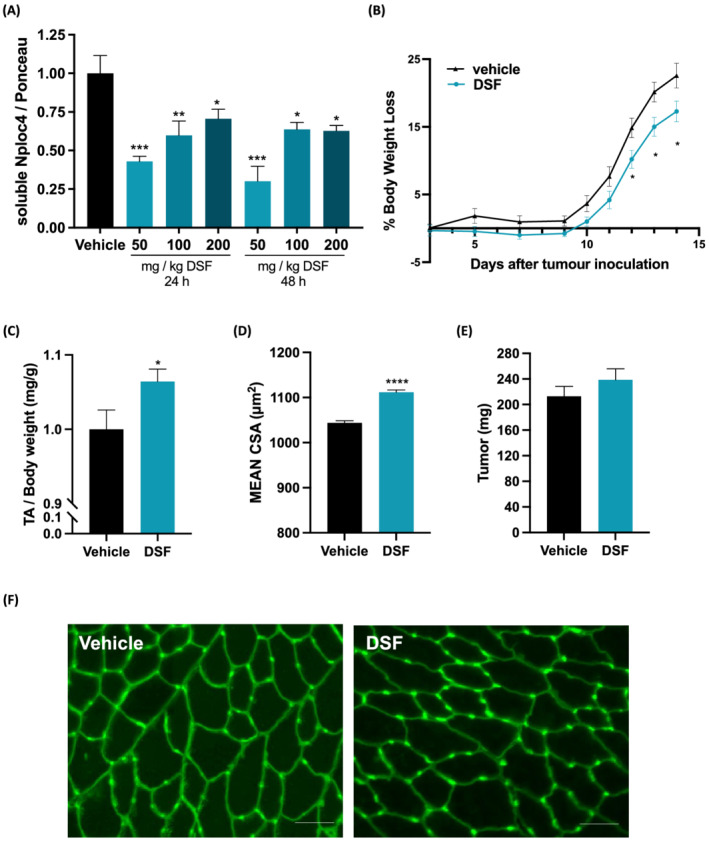
Disulfiram reduces Nploc4 in the soluble muscle fraction and preserves muscles from C26‐induced atrophy in mice. Soluble proteins from TA muscles of BALB/c mice treated with vehicle or 50, 100 or 200 mg/kg of DSF and euthanized 24 or 48 h later were analysed by WB for Nploc4. The band quantitation is shown, *n* = 2–3 (*A*). Ponceau staining was used as loading control. C26 mice (9–10 per group) were randomized to receive DSF 50 mg/kg or vehicle every 48 h from Days 3 to 13. BWL is plotted over time (*B*). TA weights at death normalized on body weights (*C*), mean cross‐sectional area (CSA) of TA (*D*) and tumour weights (*E*) are shown. A total of 16 847 fibres per group for a total of 9 mice per group was analysed. Representative images of transverse sections of fibres of TA stained for wheat‐germ agglutinin (WGA) are shown (*F*). Results are plotted as mean ± SEM. Scale bar, 50 μM. One‐way ANOVA with post‐hoc Dunnett's multiple comparison test (*A*) or two‐way ANOVA for repeated measures with additional Tukey's post‐hoc in case of interaction effect (*B*) or unpaired *t*‐test (*C*–*E*) were used, *P ≤ 0.05, **P ≤ 0.01, ***P ≤ 0.001, ****P ≤ 0.0001.

Western blot analysis showed a clear reduction in the soluble fraction of Nploc4 protein in the TA of mice treated with DSF both 24 and 48 h after the dose compared with the controls (*Figure* 8A). The lowest concentration of DSF among those lowering the levels of Nploc4 in the soluble fraction was 50 mg/kg (*Figure* 8A). To avoid undesirable side effects, we opted the 48 h schedule because we did not see any difference in Nploc4 content in the soluble fraction among 24 and 48 h (*Figure* 8A). We then inoculated BALB/c mice with C26 cells 3 days before the start of treatment with vehicle or DSF 50 mg/kg given orally every 48 h. DSF‐treated C26‐bearing mice had less body weight loss by Days 12–14 after tumour implant than vehicle‐treated mice (*Figure* 8B). Moreover, DSF clearly preserved TA muscle weight and their fibre area (*Figure* 8C, 8D, and 8F) compared with vehicle‐treated C26‐mice with no apparent effect on tumour weight at death (*Figure* 8E).

Altogether, DSF reduced Nploc4 from the soluble fraction of muscles and spared body weight and muscles from C26‐induced atrophy in mice.

## Discussion

Cancer cachexia and ALS are very different multifactorial diseases but both cause muscle atrophy. Skeletal muscle plays an essential role in the progression of both diseases because atrophy of the diaphragm and/or heart, resulting in cardio‐respiratory collapse, can be the cause of death in both disorders.^15^, ^16^

These two diseases share various similarities. The reduction in muscle mass is due in both cases to an imbalance between protein synthesis and degradation, which involves the ubiquitin‐proteasome system.^29^ Neuromuscular junction dysfunction has been described in ALS as well as, more recently, in cancer cachexia.^19^, ^20^ Moreover, when a cachexia‐inducing tumour such as LLC was injected in SOD1 knock‐out mice, a quarter of the mice died earlier than LLC‐bearing WT mice.^30^ The notion that SOD1 depletion causes premature death in LLC carriers seems a further link between cancer cachexia and ALS that is also due to mutations in SOD1. To our knowledge, the present study is the first to compare muscle wasting in these two pathologies at the molecular level, though to date there is no therapy to counteract atrophy in either.

Cancer cachexia is a heterogeneous disease. Therefore, we employed four distinct models, C26, LLC, MCG101, and RXF393, the first three in immunocompetent mice from two different strains (BALB/c and C57BL/6) and the latter in immunodeficient mice bearing a human renal carcinoma. To study ALS, mice with the SOD1^G93A^ mutation were used at two different stages of the disease. Our data indicate p97 as involved in the molecular mechanisms that induce muscle atrophy caused not only by fasting or denervation^3^ but also by cancer cachexia and ALS. Despite the beneficial effects achieved with silencing p97, this complex is involved in various processes essential for cell viability. Inhibiting this protein would be deleterious, and, in fact, p97 total knock‐out in mice is not compatible with life.^21^ However, p97 interacts with several adaptor proteins to accomplish its effects.^10^

Among various p97 cofactors, our attention was caught from Nploc4 adaptor for a number of reasons: (i) it was the most induced in TA muscle both in cancer cachexia and in ALS (*Figures* 5 and S6); (ii) the increase in gene expression was also confirmed by a raise in the protein content of Nploc4 in the atrophied muscles (*Figures* 6, S8, S9, and S10); and (iii) its induction exceeded that of p97 in atrophied muscles (ALS/cancer cachexia) (*Figures* 1, 2, 5, and 6). The Ufd1‐Nploc4 complex diverts p97 to ER‐associated protein degradation (ERAD) and to mitotic spindle disassembly after mitosis.^11^ We do not believe that Nploc4 is involved in mitotic spindle disassembly during muscle wasting because skeletal muscle is a post‐mitotic tissue, for which this mechanism may not work, but we cannot exclude that Nploc4 may be involved in mitotic spindle disassembly in other cell subtypes of muscle undergoing mitosis, like satellite cells or fibroblasts. Instead, the increase in Nploc4 in muscles during cachexia and ALS might be explained by increased ERAD, in addition to enhanced proteolysis of sarcomeric proteins. In ALS^31^ and in cancer cachexia,^32^ muscle proteins are not folded correctly, leading to an increase in the activity of the ubiquitin‐proteasome system and the relative ERAD.

To see whether the expression of Nploc4 was also altered in other conditions of muscle atrophy, we carried out an *in silico* analysis, but the adaptor was not present in previous microarrays^5^, ^33^ or proteomic analyses.^34^ Among the p97 cofactors, only Hsp90 was increased at protein level in triceps of ALS mice,^34^ in accordance with our findings in the TA (*Figure* 5E).

The only atrophic condition tested on myotubes able to increase the gene expression of Nploc4 was the treatment with 10 ng/mL TNFα/IFNγ (*Figure* S14). TNFα is one of the inflammatory cytokines induced in plasma of ALS and cachectic patients.^17^, ^18^ TNFα and IFNγ promote the phosphorylation of STAT3, which forms a complex with NF‐Kβ, and this, translocated to the nucleus, induces the expression of the genes driving atrophy.^35^ Nploc4, by promoting the degradation of the inhibitor IκBα,^36^ contributes to the activation of NF‐Kβ, and we suspect that the increased Nploc4 may favour the formation of the STAT3‐NF‐Kβ complex, thus leading to muscle atrophy also through this mechanism.

Disulfiram is a drug acting on aldehyde dehydrogenase,^26^ first approved in 1940 for the treatment of chronic alcoholism. No research on aldehyde dehydrogenase has ever been done to see whether it is implicated in muscle wasting during cancer or ALS. However, recent studies have shown that DSF inhibits the activity of Nploc4,^25^, ^26^ thus becoming an excellent drug candidate for further studies. We previously showed that actin gets degraded through the p97‐Ufd1 complex in myotubes cultured *in vitro*,^3^ and this is in accordance with data herein reported, acting Ufd1 and Nploc4 as heterodimer. DSF, therefore, has an anti‐catabolic effect by reducing protein degradation *in vitro*, ultimately sparing actin (*Figure* 7).

Colon carcinoma C26 was selected to test the anti‐cachexia effects of DSF because it causes rapid weight loss despite the small sizes of the tumour.^24^ In addition, C26 growth results in an increase in plasma TNFα, a condition that can enhance Nploc4 gene expression *in vitro* (*Figure* S14). Interestingly, although DSF has known antitumor action,^25^ the effect we found was not due to an action on the tumour, because there was no change in the size of the tumour between groups (*Figure* 8E). The protective effect in our experiments *in vitro* and *in vivo* can be explained by the fact that the DSF derivative CuET can bypass the copper transporter system and inhibit the function of p97‐Nploc4 complex in the cytosol. This may occur by releasing cupric ions under oxidative conditions, which disrupt the zinc finger motifs of Nploc4, locking the essential conformational switch of the complex.^37^

Because p97 is increased in denervation‐induced and fasting‐induced muscle atrophy,^3^ it would be interesting to measure the gene expression of Nploc4 in these two models of muscle atrophy to see whether Nploc4 can be added to the list of atrogenes. Furthermore, the increases in gene and protein expression of Nploc4 and the beneficial effect of its deletion in the TA muscle suggest that it might be useful to test DSF also in mice with ALS. The DSF active metabolite is CuET, and different copper compounds have been already successfully used to treat ALS in mice.^38^, ^39^ Finally, to study the mechanisms of the p97 complex and its adaptors during the disease, the mouse model presenting the R155H mutation, found in some patients with ALS, could be used, also to see whether—as happens for the A232E mutation present in the inclusion‐body myopathy with Paget's disease of the bone and frontotemporal dementia—such mutation increases the affinity of p97 for Nploc4.

We now believe that the p97‐Nploc4 complex plays a vital role in muscle wasting induced by cancer and ALS and targeting it could be a strategy to counteract atrophy. Inhibition of the interaction between p97 and Nploc4 shall be less deleterious for the rest of the organism than simply attenuating p97 protein.

## Conflict of interest

All authors declare they have no conflicts of interest.

## Supporting information


**Figure S1.** **The mRNA levels of p97 increase in gastrocnemius only from cancer cachexia mouse models.** The mRNA levels of *Fbxo32/atrogin 1* and *MuRF1* were evaluated in gastrocnemius (GA) of C26‐ (**A**, **E**), LLC‐ (**B**, **F**), RXF393‐ (**C**, **G**) and 4T1‐bearing mice (**D**, **H**) using qPCR. PBS‐injected mice were used as control. C26 *n* = 8–10, LLC *n* = 6–7, RXF393 *n* = 5–6 and 4 T1 n = 5–7. *Ipo8* and *GUSB* were used as housekeeping genes. The weight of the GA from PBS‐, 4 T1‐, C26‐, LLC‐ and RXF393‐mice is shown in (**I**). 4T1 *n* = 5–7, C26 *n* = 8–10, LLC *n* = 6–7 and RXF393 n = 5–6. By qPCR, the mRNA levels of *p97* in GA of 4 T1‐, C26‐, LLC‐ and RXF393‐carriers were measured and plotted as fold change over PBS‐mice (dotted line) (**L**). 4T1 *n* = 5–7, C26 *n* = 8–10, LLC *n* = 6–7 and RXF393 n = 5–6. *Ipo8* and *GUSB* were used as housekeeping genes. Results are plotted as mean ± SEM. Unpaired t‐test or Mann–Whitney test was done for each condition compared to its own PBS * *p* ≤ 0.05, ** *p* ≤ 0.01, *** *p* ≤ 0.001 and **** *p* ≤ 0.0001.
**Figure S2: The expression of p97 rises in atrophying gastrocnemius of symptomatic SOD1** ^**G93A**^ **mice.** The weights of the GA from 129/SvHsd WT or SOD1^G93A^ mice are shown in (**A**). The mRNA levels of *p97* were evaluated in GA of 129Sv SOD1^G93A^ mice at 14 and 17 weeks of age, and compared to 14 week‐old wild‐type mice (wt) using qPCR (**B**). 129/SvHsd WT mice served as Ntg controls. *n* = 5–6. *Ipo8* was used as housekeeping gene. GA of 129/SvHsd SOD1^G93A^ mice were analyzed using western blot for p97 (**C**, 14 week‐old mice; **E**, 17 week‐old mice) and the related band quantitation was plotted (**D**, **F**), *n* = 5–6. GAPDH was used as loading control. Results are plotted as mean ± SEM. One‐way ANOVA with post‐hoc Dunnett's multiple comparison test (**A**, **B**) or unpaired t‐test (**D**, **F**). * *p* ≤ 0.05, ** *p* ≤ 0.01 and *** *p* ≤ 0.001.
**Figure S3: C26‐bearing mice and SOD1** ^**G93A**^ **mice from 15 weeks of age, but not LLC‐carriers, eat less than their age‐ and sex‐matched counterparts.** Cumulative food intake is shown for ten week‐old C26‐ (**A**) and LLC‐bearing mice (**B**). PBS‐treated mice were used as controls. N (cage) = 2. Cumulative food intake for SOD1^G93A^ mice (TG) from 9 to 12 weeks of age (**C**) and from 15 to 18 weeks of age (**D**) is plotted. 129/SvHsd WT (NTG) mice were used as controls. N (cage) = 1–3. Results are plotted as mean ± SEM. Multiple unpaired t‐test, * *p* ≤ 0.05 and ** *p* ≤ 0.01.
**Figure S4: The protein content of the transcription factors Pax4 does not change in muscle with C26‐induced cachexia or ALS.** TA muscles of C26 bearing‐mice were analyzed for Pax4 by WB (**A**). The band quantitation is plotted (**B**), *n* = 3. PBS injected‐mice were used as controls and vinculin as loading control. No significant differences were found. TA muscles of 129/SvHsd SOD1^G93A^ mice were analyzed for Pax4 by WB (**C**, 14 week‐old mice; **D**, 17 week‐old mice). The band quantitation is plotted, *n* = 5–6 (**E**, **F**) and vinculin served as loading control. 129/SvHsd WT mice were used as controls. Ntg, non‐transgenic mice; Tg, transgenic mice. Results are plotted as mean ± SEM. Unpaired t‐test, no significant differences were found.
**Figure S5: Silencing p97 does not alter the fiber area of muscles of healthy mice.** Representative images of transverse section of fibers electroporated with pGIPZ empty vector as control or shRNAp97‐GFP carrying plasmid in PBS‐injected BALB/c mice (**A**) or 129/SvHsd WT mice (**C**) are shown. Scale bar, 50 μm. Frequency histograms showing the distribution of cross‐sectional areas of muscle fibers of TA transfected with the empty vector (control) or shRNAp97‐GFP carrying plasmid are reported on the right for PBS‐injected BALB/c mice (**B**) and 129/SvHsd WT mice (**D**). Two hundred and forty‐five expressing fibers were analyzed in (**A**, **B**) and 339 in (**C**, **D**). Unpaired t‐test or Mann–Whitney test, no significant differences were found.
**Figure S6: Among the p97 cofactors, Nploc4 is the one most induced in atrophying gastrocnemius from either cancer cachexia models.** By qPCR, the mRNA levels of *p97* adaptors in GA of C26‐ (**A**), LLC‐ (**B**) and RXF393‐mice (**C**) was measured and plotted as fold change over PBS‐mice (dotted line). C26 *n* = 8–9, LLC *n* = 5–6 and RXF393 *n* = 6. *Ipo8 and Gusb* served as housekeeping genes for (**A**‐**C**). Nploc4 expression level was evaluated in non‐cachectic GA muscles of 4 T1‐mice using qPCR (**D**) *n* = 5–7. PBS‐mice were used as controls. *Gusb* served as housekeeping gene. All results are plotted as mean ± SEM. Unpaired t‐test or Mann–Whitney test, * *p* ≤ 0.05, ** *p* ≤ 0.01 and **** *p* ≤ 0.0001.
**Figure S7: The mRNA levels of *Nploc4* and *p97* increase in GA muscles of MCG101‐bearing mice.** The mRNA levels of *Nploc4* (**A**), *p97* (**B**), *MuRF1* (**C**) and *Fbxo32/atrogin 1* (**D**) were evaluated in GA of MCG101‐bearing mice using qPCR. PBS‐injected mice were used as control. *n* = 4–5. *Ipo8* was used as housekeeping gene. All results are plotted as mean ± SEM. Unpaired t‐test or Mann–Whitney test, * *p* ≤ 0.05 and ** *p* ≤ 0.01.
**Figure S8: Nploc4 and MuRF1 rise in atrophying gastrocnemius of symptomatic SOD1** ^**G93A**^ **mice.** GA muscles of 129/SvHsd SOD1^G93A^ mice of 17 weeks of age were analyzed using western blot for Nploc4, p47, Ufd1 and MuRF1 (**A**) and the related band quantitation was plotted (**B**‐**E**). *n* = 5–6. GAPDH was used as loading control for Nploc4, p47 and Ufd1, while vinculin for MuRF1. 129/SvHsd WT mice were used as controls. Results are plotted as mean ± SEM. Unpaired t‐test, * *p* ≤ 0.05 and *** *p* ≤ 0.001.
**Figure S9: In atrophying gastrocnemius of RXF393‐ and LLC‐bearing mice, the protein content of Nploc4 tends to increase at times when MuRF1 rises.** GA muscles of RXF393‐bearing mice were analyzed using western blot for Nploc4, p47, Ufd1 and MuRF1 (**A**) and the related band quantitation was plotted (**B**‐**E**), *n* = 5–6. Vinculin was used as internal loading control. PBS‐injected mice were used as controls. GA muscles of LLC‐bearing mice were analyzed using western blot for Nploc4, Ufd1 and MuRF1 (**F**) and the related band quantitation was plotted, (**G**‐**I**), *n* = 6–7 Coomassie blue staining was used to normalize the data. PBS‐injected mice were used as controls. Results are plotted as mean ± SEM. Unpaired t‐test, *** *p* ≤ 0.001.
**Figure S10: The protein contents of either p97 or Nploc4 do not rise in muscles of 4 T1‐bearing mice.** GA muscles of 4T1‐bearing mice were analyzed using western blot for p97, Nploc4, p47 and Ufd1 (**A**) and the related band quantitation was plotted (**B‐E**), *n* = 5–9. Vinculin was used as loading control. PBS‐injected mice were used as controls. Results are plotted as mean ± SEM. Unpaired t‐test, no significant differences were found.
**Figure S11: CRISPR/Cas9 Nploc4 plasmid carries a guide that can edit the target gene.** Agarose gel of the T7E1 cleavage assay of CRISPR/Cas9 Nploc4 plasmid transfected in C2C12 cells for 72 h is shown. CRISPR/Cas9 Ctrl is an empty vector, while “Ctrl” is the manufacturer control of the reaction.
**Figure S12: Silencing Nploc4 does not alter the fiber area of muscles of healthy mice.** Representative images of transverse section of fibers electroporated with empty vector (control) or CRISPR/Cas9 Nploc4 carrying plasmid (CC9 Nploc4) in PBS‐injected BALB/c (**A**) or 129/SvHsd (**C**) mice are shown. Scale bar, 50 μm. Frequency histograms showing the distribution of cross‐sectional areas of muscle fibers of TA transfected with the control) or CC9 Nploc4 vector are reported on the right (**B**, **D**). Two hundred eighty‐eight expressing fibers were analyzed in (**A**, **B**) and 349 in (**C**, **D**). Unpaired t‐test or Mann–Whitney test, no significant differences were found.
**Figure S13: Twenty four hours‐treatment with 10 μM, but not 0.1 or 1 μM, disulfiram reduces the viability of cultured myotubes.** The viability of myotubes treated for 24 h with disulfiram (DSF) ranging from 0.1 to 10 μM was measured with SRB assays and expressed as percentage of the corresponding vehicle‐treated cells, as controls, *n* = 4–5. One‐way ANOVA with post‐hoc Dunnett's multiple comparison test, * *p* ≤ 0.05 and **** *p* ≤ 0.0001.
**Figure S14: The mRNA levels of Nploc4 are induced in atrophying myotubes treated with TNFα/IFNγ.** C2C12 myotubes on the fourth day of differentiation were treated for 24 h or 48 h with vehicle or 10 μM dexamethasone, 10 ng/mL IFNγ / TNFα, 10 ng/mL or 100 ng/mL IL‐6, 10 ng/mL Activin A. For caFoXO1 and caFoXO3, myotubes were infected with adenovirus expressing these proteins, as in (1). For the treatment with C26 supernatants or transwell system refer to (2). The mRNA levels of Nploc4 was measured by qPCR in myotubes treated with different atrophying stimuli and plotted as fold change over vehicle (dotted line). *n* = 2–6. *Ipo8* and *TBP* served as housekeeping (HK) genes. tw = transwell; SN = supernatant; ca = constitutively active. Results are plotted as mean ± SEM. Unpaired t‐test or Mann–Whitney test, * *p* ≤ 0.05.

## References

[1] Bodnar N , Rapoport T . Toward an understanding of the Cdc48/p97 ATPase. F1000Research 2017;6:1318. 28815021 10.12688/f1000research.11683.1 PMC5543420

[2] Ramadan K , Bruderer R , Spiga FM , Popp O , Baur T , Gotta M , et al. Cdc48/p97 promotes reformation of the nucleus by extracting the kinase Aurora B from chromatin. Nature 2007;450:1258–1262. 18097415 10.1038/nature06388

[3] Piccirillo R , Goldberg AL . The p97/VCP ATPase is critical in muscle atrophy and the accelerated degradation of muscle proteins. EMBO J 2012;31:3334–3350. 22773186 10.1038/emboj.2012.178 PMC3411080

[4] Ye Y , Meyer HH , Rapoport TA . The AAA ATPase Cdc48/p97 and its partners transport proteins from the ER into the cytosol. Nature 2001;414:652–656. 11740563 10.1038/414652a

[5] Lecker SH , Jagoe RT , Gilbert A , Gomes M , Baracos V , Bailey J , et al. Multiple types of skeletal muscle atrophy involve a common program of changes in gene expression. FASEB J 2004;18:39–51. 14718385 10.1096/fj.03-0610com

[6] Volodin A , Kosti I , Goldberg AL , Cohen S . Myofibril breakdown during atrophy is a delayed response requiring the transcription factor PAX4 and desmin depolymerization. Proc Natl Acad Sci U S A 2017;114:E1375–E1384. 28096335 10.1073/pnas.1612988114 PMC5338431

[7] Watts GDJ , Wymer J , Kovach MJ , Mehta SG , Mumm S , Darvish D , et al. Inclusion body myopathy associated with Paget disease of bone and frontotemporal dementia is caused by mutant valosin‐containing protein. Nat Genet 2004;36:377–381. 15034582 10.1038/ng1332

[8] Johnson JO , Mandrioli J , Benatar M , Abramzon Y , van Deerlin V , Trojanowski JQ , et al. Exome sequencing reveals VCP mutations as a cause of familial ALS. Neuron 2010;68:857–864. 21145000 10.1016/j.neuron.2010.11.036 PMC3032425

[9] Kimonis VE , Fulchiero E , Vesa J , Watts G . VCP disease associated with myopathy, Paget disease of bone and frontotemporal dementia: review of a unique disorder. Biochim Biophys Acta 2008;1782:744–748. 18845250 10.1016/j.bbadis.2008.09.003

[10] Jentsch S , Rumpf S . Cdc48 (p97): a ‘molecular gearbox’ in the ubiquitin pathway? Trends Biochem Sci 2007;32:6–11. 17142044 10.1016/j.tibs.2006.11.005

[11] Meyer HH , Shorter JG , Seemann J , Pappin D , Warren G . A complex of mammalian ufd1 and npl4 links the AAA‐ATPase, p97, to ubiquitin and nuclear transport pathways. EMBO J 2000;19:2181–2192. 10811609 10.1093/emboj/19.10.2181 PMC384367

[12] Lokireddy S , Wijesoma IW , Sze SK , McFarlane C , Kambadur R , Sharma M . Identification of atrogin‐1‐targeted proteins during the myostatin‐induced skeletal muscle wasting. Am J Physiol Cell Physiol 2012;303:C512–C529. 22673621 10.1152/ajpcell.00402.2011

[13] Bodine SC , Latres E , Baumhueter S , Lai VK , Nunez L , Clarke BA , et al. Identification of ubiquitin ligases required for skeletal muscle atrophy. Science 2001;294:1704–1708. 11679633 10.1126/science.1065874

[14] Leger B , Vergani L , Soraru G , Hespel P , Derave W , Gobelet C , et al. Human skeletal muscle atrophy in amyotrophic lateral sclerosis reveals a reduction in Akt and an increase in atrogin‐1. FASEB J 2006;20(3):583–585. 16507768 10.1096/fj.05-5249fje

[15] Houten L , Reilley AA . An investigation of the cause of death from cancer. J Surg Oncol 1980;13:111–116. 7359917 10.1002/jso.2930130205

[16] Oskarsson B , Gendron TF , Staff NP . Amyotrophic lateral sclerosis: an update for 2018. Mayo Clin Proc 2018;93:1617–1628. 30401437 10.1016/j.mayocp.2018.04.007

[17] Tortelli R , Zecca C , Piccininni M , Benmahamed S , Dell'Abate MT , Barulli MR , et al. Plasma inflammatory cytokines are elevated in ALS. Front Neurol 2020;11:552295. 33281700 10.3389/fneur.2020.552295 PMC7691268

[18] Argilés JM , Busquets S , Toledo M , López‐Soriano FJ . The role of cytokines in cancer cachexia. Curr Opin Support Palliat Care 2009;3:263–268. 19713854 10.1097/SPC.0b013e3283311d09

[19] Huot JR , Pin F , Bonetto A . Muscle weakness caused by cancer and chemotherapy is associated with loss of motor unit connectivity. Am J Cancer Res 2021;11:2990–3001. 34249440 PMC8263661

[20] Sartori R , Hagg A , Zampieri S , Armani A , Winbanks CE , Viana LR , et al. Perturbed BMP signaling and denervation promote muscle wasting in cancer cachexia. Sci Transl Med 2021;13:eaay9592. 34349036 10.1126/scitranslmed.aay9592

[21] Müller JMM , Deinhardt K , Rosewell I , Warren G , Shima DT . Targeted deletion of p97 (VCP/CDC48) in mouse results in early embryonic lethality. Biochem Biophys Res Commun 2007;354:459–465. 17239345 10.1016/j.bbrc.2006.12.206

[22] Pretto F , Ghilardi C , Moschetta M , Bassi A , Rovida A , Scarlato V , et al. Sunitinib prevents cachexia and prolongs survival of mice bearing renal cancer by restraining STAT3 and MuRF‐1 activation in muscle. Oncotarget 2015;6:3043–3054. 25460504 10.18632/oncotarget.2812 PMC4413636

[23] Nardo G , Trolese MC , Tortarolo M , Vallarola A , Freschi M , Pasetto L , et al. New insights on the mechanisms of disease course variability in ALS from mutant SOD1 mouse models. Brain Pathol 2016;26:237–247. 26780365 10.1111/bpa.12351 PMC8029191

[24] Re Cecconi AD , Forti M , Chiappa M , Zhu Z , Zingman LV , Cervo L , et al. Musclin, a myokine induced by aerobic exercise, retards muscle atrophy during cancer cachexia in mice. Cancer 2019;11:1541. 10.3390/cancers11101541 PMC6826436 31614775

[25] Majera D , Skrott Z , Chroma K , Merchut‐Maya JM , Mistrik M , Bartek J . Targeting the NPL4 adaptor of p97/VCP segregase by disulfiram as an emerging cancer vulnerability evokes replication stress and DNA damage while silencing the ATR pathway. Cell 2020;9. 10.3390/cells9020469 PMC7072750 32085572

[26] Skrott Z , Mistrik M , Andersen KK , Friis S , Majera D , Gursky J , et al. Alcohol‐abuse drug disulfiram targets cancer via p97 segregase adaptor NPL4. Nature 2017;552:194–199. 29211715 10.1038/nature25016 PMC5730499

[27] Moresi V , Williams AH , Meadows E , Flynn JM , Potthoff MJ , McAnally J , et al. Myogenin and class II HDACs control neurogenic muscle atrophy by inducing E3 ubiquitin ligases. Cell 2010;143:35–45. 20887891 10.1016/j.cell.2010.09.004 PMC2982779

[28] Ham DJ , Börsch A , Lin S , Thürkauf M , Weihrauch M , Reinhard JR , et al. The neuromuscular junction is a focal point of mTORC1 signaling in sarcopenia. Nat Commun 2020;11:4510. 32908143 10.1038/s41467-020-18140-1 PMC7481251

[29] Lecker SH , Goldberg AL , Mitch WE . Protein degradation by the ubiquitin‐proteasome pathway in normal and disease states. J Am Soc Nephrol JASN 2006;17:1807–1819. 16738015 10.1681/ASN.2006010083

[30] Brown JL , Lawrence MM , Ahn B , Kneis P , Piekarz KM , Qaisar R , et al. Cancer cachexia in a mouse model of oxidative stress. J Cachexia Sarcopenia Muscle 2020;11:1688–1704. 32918528 10.1002/jcsm.12615 PMC7749559

[31] Prell T , Lautenschläger J , Weidemann L , Ruhmer J , Witte OW , Grosskreutz J . Endoplasmic reticulum stress is accompanied by activation of NF‐κB in amyotrophic lateral sclerosis. J Neuroimmunol 2014;270:29–36. 24666819 10.1016/j.jneuroim.2014.03.005

[32] Roy A , Kumar A . ER stress and unfolded protein response in cancer cachexia. Cancer 2019;11. 10.3390/cancers11121929 PMC6966641 31817027

[33] Sacheck JM , Hyatt J‐PK , Raffaello A , Thomas Jagoe R , Roy RR , Reggie Edgerton V , et al. Rapid disuse and denervation atrophy involve transcriptional changes similar to those of muscle wasting during systemic diseases. FASEB J 2007;21:140–155. 17116744 10.1096/fj.06-6604com

[34] Capitanio D , Vasso M , Ratti A , Grignaschi G , Volta M , Moriggi M , et al. Molecular signatures of amyotrophic lateral sclerosis disease progression in hind and forelimb muscles of an SOD1(G93A) mouse model. Antioxid Redox Signal 2012;17:1333–1350. 22563797 10.1089/ars.2012.4524 PMC3437050

[35] Ma JF , Sanchez BJ , Hall DT , Tremblay A‐MK , Di Marco S , Gallouzi I‐E . STAT3 promotes IFNγ/TNFα‐induced muscle wasting in an NF‐κB‐dependent and IL‐6‐independent manner. EMBO Mol Med 2017;9:622–637. 28264935 10.15252/emmm.201607052 PMC5412921

[36] Li J‐M , Wu H , Zhang W , Blackburn MR , Jin J . The p97‐UFD1L‐NPL4 protein complex mediates cytokine‐induced IκBα proteolysis. Mol Cell Biol 2014;34:335–347. 24248593 10.1128/MCB.01190-13 PMC3911508

[37] Pan M , Zheng Q , Yu Y , Ai H , Xie Y , Zeng X , et al. Seesaw conformations of Npl4 in the human p97 complex and the inhibitory mechanism of a disulfiram derivative. Nat Commun 2021;12:121. 33402676 10.1038/s41467-020-20359-x PMC7785736

[38] Roberts BR , Lim NKH , McAllum EJ , Donnelly PS , Hare DJ , Doble PA , et al. Oral treatment with Cu (II)(atsm) increases mutant SOD1 in vivo but protects motor neurons and improves the phenotype of a transgenic mouse model of amyotrophic lateral sclerosis. J Neurosci 2014;34:8021–8031. 24899723 10.1523/JNEUROSCI.4196-13.2014 PMC6608261

[39] Williams JR , Trias E , Beilby PR , Lopez NI , Labut EM , Bradford CS , et al. Copper delivery to the CNS by CuATSM effectively treats motor neuron disease in SOD(G93A) mice co‐expressing the Copper‐Chaperone‐for‐SOD. Neurobiol Dis 2016;89:1–9. 26826269 10.1016/j.nbd.2016.01.020 PMC4785045

[40] von Haehling S , Morley JE , Coats AJS , Anker SD . Ethical guidelines for publishing in the Journal of Cachexia, Sarcopenia and Muscle: update 2021. J Cachexia Sarcopenia Muscle 2021;12:2259–2261. 34904399 10.1002/jcsm.12899 PMC8718061

