# Supplementary material for: The p97‐Nploc4 ATPase complex plays a role in muscle atrophy during cancer and amyotrophic lateral sclerosis
Source: J Cachexia Sarcopenia Muscle. 2022 May 25;13(4):2225–41. doi: 10.1002/jcsm.13011 (PMC9397562; doi:10.1002/jcsm.13011)
Supplement: Supplementary file 1 — Figure S1. The mRNA levels of p97 increase in gastrocnemius only from cancer cachexia mouse models. The mRNA levels of Fbxo32/atrogin 1 and MuRF1 were evaluated in gastrocnemius (GA) of C26‐ (A, E), LLC‐ (B, F), RXF393‐ (C, G) and 4T1‐bearing mice (D, H) using qPCR. PBS‐injected mice were used as control. C26 n = 8–10, LLC n = 6–7, RXF393 n = 5–6 and 4 T1 n = 5–7. Ipo8 and GUSB were used as housekeeping genes. The weight of the GA from PBS‐, 4 T1‐, C26‐, LLC‐ and RXF393‐mice is shown in (I). 4T1 n = 5–7, C26 n = 8–10, LLC n = 6–7 and RXF393 n = 5–6. By qPCR, the mRNA levels of p97 in GA of 4 T1‐, C26‐, LLC‐ and RXF393‐carriers were measured and plotted as fold change over PBS‐mice (dotted line) (L). 4T1 n = 5–7, C26 n = 8–10, LLC n = 6–7 and RXF393 n = 5–6. Ipo8 and GUSB were used as housekeeping genes. Results are plotted as mean ± SEM. Unpaired t‐test or Mann–Whitney test was done for each condition compared to its own PBS * p ≤ 0.05, ** p ≤ 0.01, *** p ≤ 0.001 and **** p ≤ 0.0001. Figure S2: The expression of p97 rises in atrophying gastrocnemius of symptomatic SOD1 G93A mice. The weights of the GA from 129/SvHsd WT or SOD1G93A mice are shown in (A). The mRNA levels of p97 were evaluated in GA of 129Sv SOD1G93A mice at 14 and 17 weeks of age, and compared to 14 week‐old wild‐type mice (wt) using qPCR (B). 129/SvHsd WT mice served as Ntg controls. n = 5–6. Ipo8 was used as housekeeping gene. GA of 129/SvHsd SOD1G93A mice were analyzed using western blot for p97 (C, 14 week‐old mice; E, 17 week‐old mice) and the related band quantitation was plotted (D, F), n = 5–6. GAPDH was used as loading control. Results are plotted as mean ± SEM. One‐way ANOVA with post‐hoc Dunnett's multiple comparison test (A, B) or unpaired t‐test (D, F). * p ≤ 0.05, ** p ≤ 0.01 and *** p ≤ 0.001. Figure S3: C26‐bearing mice and SOD1 G93A mice from 15 weeks of age, but not LLC‐carriers, eat less than their age‐ and sex‐matched counterparts. Cumulative food intake is shown for ten week‐old C [file JCSM-13-2225-s001.docx]

**SUPPLEMENTARY FIGURE LEGENDS**

**Supplementary Figure 1: The mRNA levels of p97 increase in gastrocnemius only from cancer cachexia mouse models.** The mRNA levels of *Fbxo32/atrogin 1* and *MuRF1* were evaluated in gastrocnemius (GA) of C26- (**A**, **E**), LLC- (**B**, **F**), RXF393- (**C**, **G**) and 4T1-bearing mice (**D**, **H**) using qPCR. PBS-injected mice were used as control. C26 n=8-10, LLC n=6-7, RXF393 n=5-6 and 4T1 n=5-7. *Ipo8*, *TBP* and *GUSB* were used as housekeeping genes. The weight of the GA from PBS-, 4T1-, C26-, LLC- and RXF393-mice is shown in (**I**). 4T1 n=5-7, C26 n=8-10, LLC n=6-7 and RXF393 n=5-6. By qPCR, the mRNA levels of *p97* in GA of 4T1-, C26-, LLC- and RXF393-carriers were measured and plotted as fold change over PBS-mice (dotted line) (**L**). 4T1 n=5-7, C26 n=8-10, LLC n=6-7 and RXF393 n=5-6. *Ipo8*, *TBP* and *GUSB* were used as housekeeping genes. Results are plotted as mean ± SEM. Unpaired t-test or Mann-Whitney test was done for each condition compared to its own PBS * p ≤ 0.05, ** p ≤ 0.01, *** p ≤ 0.001 and **** p ≤ 0.0001.

**Supplementary Figure 2: The expression of p97 rises in atrophying gastrocnemius of symptomatic SOD1^G93A^ mice.** The weights of the GA from 129/SvHsd WT or SOD1^G93A^ mice are shown in (**A**). The mRNA levels of *p97* were evaluated in GA of 129Sv SOD1^G93A^ mice at 14 and 17 weeks of age, and compared to 14 week-old wild-type mice (wt) using qPCR (**B**). 129/SvHsd WT mice served as Ntg controls. n=5-6. *Ipo8* was used as housekeeping gene. GA of 129/SvHsd SOD1^G93A^ mice were analyzed using western blot for p97 (**C**, 14 week-old mice; **E**, 17 week-old mice) and the related band quantitation was plotted (**D**, **F**), n=5-6. GAPDH was used as loading control. Results are plotted as mean ± SEM. One-way ANOVA with post-hoc Dunnett’s multiple comparison test (**A**, **B**) or unpaired t-test (**D**, **F**). * p ≤ 0.05, ** p ≤ 0.01 and *** p ≤ 0.001.

**Supplementary Figure 3: C26-bearing mice and SOD1^G93A^ mice from 15 weeks of age, but not LLC-carriers, eat less than their age- and sex-matched counterparts.** Cumulative food intake is shown for ten week-old C26- (**A**) and LLC-bearing mice (**B**). PBS-treated mice were used as controls. N (cage)=2. Cumulative food intake for SOD1^G93A^ mice (TG) from 9 to 12 weeks of age (**C**) and from 15 to 18 weeks of age (**D**) is plotted. 129/SvHsd WT (NTG) mice were used as controls. N (cage)=1-3. Results are plotted as mean ± SEM. Multiple unpaired t-test, * p ≤ 0.05 and ** p ≤ 0.01.

**Supplementary Figure 4: The protein content of the transcription factors Pax4 does not change in muscle with C26-induced cachexia or ALS.** TA muscles of C26 bearing-mice were analyzed for Pax4 by WB (**A**). The band quantitation is plotted (**B**), n=3. PBS injected-mice were used as controls and vinculin as loading control. Results are plotted as mean ± SEM. One-way ANOVA with post-hoc Dunnett’s multiple comparison test (**B**). No significant differences were found. TA muscles of 129/SvHsd SOD1^G93A^ mice were analyzed for Pax4 by WB (**C**, 14 week-old mice; **D**, 17 week-old mice). The band quantitation is plotted, n=5-6 (**E**, **F**) and vinculin served as loading control. 129/SvHsd WT mice were used as controls. Ntg, non-transgenic mice; Tg, transgenic mice. Results are plotted as mean ± SEM. Unpaired t-test, no significant differences were found.

**Supplementary Figure 5: Silencing p97 does not alter the fiber area of muscles of healthy mice.** Representative images of transverse section of fibers electroporated with pGIPZ empty vector as control or shRNAp97-GFP carrying plasmid in PBS-injected BALB/c mice (**A**) or 129/SvHsd WT mice (**C**) are shown. Scale bar, 50 μm. Frequency histograms showing the distribution of cross-sectional areas of muscle fibers of TA transfected with the empty vector (control) or shRNAp97-GFP carrying plasmid are reported on the right for PBS-injected BALB/c mice (**B**) and 129/SvHsd WT mice (**D**). Two hundred and forty-five expressing fibers were analyzed in (**A**, **B**) and 339 in (**C**, **D**). Unpaired t-test or Mann-Whitney test, no significant differences were found.

**Supplementary Figure 6: Among the p97 cofactors, Nploc4 is the one most induced in atrophying gastrocnemius from either cancer cachexia models.** By qPCR, the mRNA levels of *p97* adaptors in GA of C26- (**A**), LLC- (**B**) and RXF393-mice (**C**) was measured and plotted as fold change over PBS-mice (dotted line). C26 n=8-9, LLC n=5-6 and RXF393 n=6. *Ipo8 and Gusb* served as housekeeping gene for (**A**, **B** and **C**). Nploc4 expression level was evaluated in non-cachectic GA muscles of 4T1-mice using qPCR (**D**) n=5-7. PBS-mice were used as controls. *Gusb* served as housekeeping gene. All results are plotted as mean ± SEM. Unpaired t-test or Mann-Whitney test,  *p* ≤ 0.05, ** *p* ≤ 0.01 and **** *p* ≤ 0.0001.

**Supplementary Figure 7: The mRNA levels of *Nploc4* and *p97* increase in GA muscles of MCG101-bearing mice.** The mRNA levels of *Nploc4* (**A**), *p97* (**B**), *MuRF1* (**C**) and *Fbxo32/atrogin* *1* (**D**) were evaluated in GA of MCG101-bearing mice using qPCR. PBS-injected mice were used as control. n=4-5. *Ipo8* was used as housekeeping gene. All results are plotted as mean ± SEM. Unpaired t-test or Mann-Whitney test, * *p* ≤ 0.05 and ** *p* ≤ 0.01.

**Supplementary figure 8: Nploc4 and MuRF1 rise in atrophying gastrocnemius of symptomatic SOD1^G93A^ mice.** GA muscles of 129/SvHsd SOD1^G93A^ mice of 17 weeks of age were analyzed using western blot for Nploc4, p47, Ufd1 and MuRF1 (**A**) and the related band quantitation was plotted (**B**-**E**). n=5-6. GAPDH was used as loading control for Nploc4, p47 and Ufd1, while vinculin for MuRF1. 129/SvHsd WT mice were used as controls. Results are plotted as mean ± SEM. Unpaired t-test, * *p* ≤ 0.05 and *** *p* ≤ 0.001.

**Supplementary Figure 9: In atrophying gastrocnemius of RXF393- and LLC-bearing mice, the protein content of Nploc4 tends to increase at times when MuRF1 rises.** GA muscles of RXF393-bearing mice were analyzed using western blot for Nploc4, p47, Ufd1 and MuRF1 (**A**) and the related band quantitation was plotted (**B**-**E**), n=5-6. Vinculin was used as internal loading control. PBS-injected mice were used as controls. GA muscles of LLC-bearing mice were analyzed using western blot for Nploc4, Ufd1 and MuRF1 (**F**) and the related band quantitation was plotted, (**G**-**I**), n=6-7 Coomassie blue staining was used to normalize the data. PBS-injected mice were used as controls. Results are plotted as mean ± SEM. Unpaired t-test, *** *p* ≤ 0.001.

**Supplementary Figure 10: The protein content of either p97 or Nploc4 do not rise in muscles of 4T1-bearing mice.** GA muscles of 4T1-bearing mice were analyzed using western blot for p97, Nploc4, p47 and Ufd1 (**A**) and the related band quantitation was plotted (**B-E**), n = 5-9. Vinculin was used as loading control. PBS-injected mice were used as controls. Results are plotted as mean ± SEM. Unpaired t-test, no significant differences were found.

**Supplementary Figure 11: Crispr/CAS9 Nploc4 plasmid carries a guide that can edit the target gene.** Agarose gel of the T7E1 cleavage assay of Crispr/CAS9 Nploc4 plasmid transfected in C2C12 cells for 72h is shown. Crispr/CAS9 Ctrl is an empty vector, while “Ctrl” is the manufacturer control of the reaction.

**Supplementary Figure 12: Silencing Nploc4 does not alter the fiber area of muscles of healthy mice.** Representative images of transverse section of fibers electroporated with empty vector (control) or Crispr/CAS9 Nploc4 carrying plasmid (CC9 Nploc4) in PBS-injected BALB/c (**A**) or 129/SvHsd (**C**) mice are shown. Scale bar, 50 μm. Frequency histograms showing the distribution of cross-sectional areas of muscle fibers of TA transfected with the control) or CC9 Nploc4 vector are reported on the right (**B**, **D**). Two hundred eighty-eight expressing fibers were analyzed in (**A**, **B**) and 349 in (**C**, **D**). Unpaired t-test or Mann-Whitney test, no significant differences were found.

**Supplementary Figure 13: Twenty four hours-treatment with 10 μM, but not 0.1 or 1 μM, disulfiram reduces the viability of cultured myotubes.** The viability of myotubes treated for 24h with disulfiram (DSF) ranging from 0.1 to 10 µM was measured with SRB assays and expressed as percentage of the corresponding vehicle-treated cells, as controls, n=4-5. One-way ANOVA with post-hoc Dunnett’s multiple comparison test, * p ≤ 0.05 and **** p ≤ 0.0001.

**Supplementary Figure 14: The mRNA levels of Nploc4 are induced in atrophying myotubes treated with TNFα/IFNγ.** C2C12 myotubes on the fourth day of differentiation were treated for 24h or 48h with vehicle or 10 μM dexamethasone, 10 ng/mL IFNγ / TNFα, 10 ng/mL or 100 ng/mL IL-6, 10 ng/mL Activin A. For caFoXO1 and caFoXO3, myotubes were infected with adenovirus expressing these proteins, as in (1). For the treatment with C26 supernatants or transwell system refer to (2). The mRNA levels of Nploc4 was measured by qPCR in myotubes treated with different atrophying stimuli and plotted as fold change over vehicle (dotted line). n=2-6. *Ipo8* and *TBP* served as housekeeping (HK) genes. tw= transwell; SN= supernatant; ca= constitutively active. Results are plotted as mean ± SEM. Unpaired t-test or Mann-Whitney test, * p ≤ 0.05.

**SUPPLEMENTARY MATERIAL AND METHODS**

**T7 assay**

To investigate whether the Crispr/CAS9 (CC9) Nploc4 plasmid specifically edits the *Nploc4* gene, we employed the GeneArt Genomic Cleavage Detection kit (Invitrogen). C2C12 myoblasts were transfected for 72h with the CC9 Nploc4 or CC9 control plasmid. After cell lysis and DNA extraction, the editing regions were amplified by PCR and the amplification product was loaded onto a 2% agarose gel.

**Western Blotting**

We employed anti-Pax4 (Abcam), anti-MuRF1 antibodies (kindly donated by Prof. Alfred L. Goldberg from Harvard Medical School, Boston, USA) and Coomassie Blue staining (LC6065, Thermo Fisher Scientific).

**Cell culture**

The viability of myotubes treated for 24h with disulfiram (DSF, Sigma, St. Louis, MO, USA) from 0.1 to 10 µM was measured with Sulforhodamine B (SRB) assay (Sigma), using Dimethylsulfoxide (DMSO) as vehicle. For Figure S14, we used Activin A (PrepoTech, Hamburg, Germany) and IL6 (Bio-Techne s.r.l., Milan, Italy). MCG101 is a sarcoma cell line, grown in McCoy’s 5A medium with 10% FBS and 2 mM L-glutamine at 37°C with 5% CO_2_. MCG101 cells were shared by Prof. Anders Blomqvist (Linköping University, Sweden).

**Food Intake**

Food intake was measured on a per-cage basis. The mean cumulative food intake shown is the sum over time of all the food consumed on average by the animals during the whole experimental or the indicated period for ALS.

**SUPPLEMENTARY REFERENCES**

1. Re Cecconi AD, Forti M, Chiappa M, Zhu Z, Zingman LV, Cervo L *et al.* Musclin, A Myokine Induced by Aerobic Exercise, Retards Muscle Atrophy During Cancer Cachexia in Mice. *Cancers* 2019;**11**:1541.
2. Aquila G, Re Cecconi AD, Forti M, Frapolli R, Bello E, Novelli D *et al.* Trabectedin and Lurbinectedin Extend Survival of Mice Bearing C26 Colon Adenocarcinoma, without Affecting Tumor Growth or Cachexia. *Cancers* 2020;**12**.
